# Challenge-based learning in blended education: a meta-analysis of deep learning outcomes

**DOI:** 10.3389/fpsyg.2026.1939274

**Published:** 2026-09-23

**Authors:** Zhonghua Sun, Siyu Chen, Mei Bie

**Affiliations:** 1 Institute of Education, Changchun Normal University, Changchun, China; 2 College of Computer Science and Technology, Jilin University, Changchun, China

**Keywords:** blended education, challenge-based learning, deep learning, offline education, technological integration

## Abstract

Challenge-based learning (CBL) has gained increasing attention in higher education as a student-centered approach within blended and integrated learning, yet its effectiveness in fostering deep learning and the conditions under which it is most effective remain insufficiently established. This meta-analysis synthesized evidence from 22 experimental and quasi-experimental studies conducted in higher education to estimate the overall effect of CBL on deep learning and examine potential moderators, including discipline, grade level, class size, teaching duration, technological support, learning environment, and knowledge type. CBL demonstrated a statistically significant overall positive effect on deep learning [*g* = 0.792, 95% CI (0.549, 1.035), *p* < 0.001]. In the dependency-adjusted joint Robust Variance Estimation (RVE) meta-regression, disciplinary domain (*p* = 0.024) and intervention duration (*p* = 0.038) were significantly associated with variation in effect sizes as independent moderators, with larger effects observed in STEM contexts and longer implementation durations (>12 weeks). Separate subgroup analyses indicated differences in effect sizes across academic levels and class-size categories; however, these findings were not interpreted as independent moderator effects unless supported by the joint model. Teaching duration, environment (online, offline, or blended), and knowledge type showed no statistically significant moderation. While separate univariate subgroup analyses indicated significant moderation across academic levels (*p* = 0.002) and class-size categories (*p* = 0.029), these variables failed to retain independent moderation status in the joint model. Although some subgroup patterns suggested differences across academic levels and learning environments, the available evidence did not establish a linear or monotonic relationship between academic seniority and CBL effectiveness. Overall, CBL is an effective strategy for promoting deep learning, especially when supported by digital infrastructure and implemented in disciplines and contexts aligned with its active, inquiry-driven model. Future implementations should optimize technological integration and pedagogical alignment to maximize impact.

## 1 Introduction

As higher education systems increasingly emphasize the development of transferable competencies for complex and rapidly changing professional environments, educational researchers have become increasingly concerned with how instructional approaches can promote meaningful and sustainable learning. In this study, deep learning refers to a pedagogical construct rather than the computational concept used in artificial intelligence. It encompasses learners' ability to engage in higher-order cognitive processes, including critical thinking, problem-solving, knowledge integration, reflection, and self-regulated learning. Such competencies are widely recognized as essential for addressing complex real-world problems and adapting to evolving social and professional contexts (Rahimi, 2024; Van Laar et al., 2020). The growing emphasis on deep learning reflects a broader shift from knowledge transmission toward knowledge construction. Traditional instructional approaches often focus on the acquisition and reproduction of factual knowledge, whereas contemporary educational frameworks increasingly prioritize students' ability to apply knowledge in authentic, ill-structured situations. Consequently, educational systems worldwide have highlighted deep learning as a key outcome of twenty-first century education (González-Pérez and Ramírez-Montoya, 2022; Van Laar et al., 2019).

Among the various pedagogical approaches proposed to foster deep learning, CBL has received increasing attention. Originating from student-centered and inquiry-oriented educational traditions, CBL engages learners in identifying, investigating, and addressing authentic challenges that are socially relevant and contextually meaningful (Bilbao-Goyoaga et al., 2023; Johnson and Brown, 2011; Portuguez Castro and Gomez Zermeno, 2020). Rather than emphasizing the passive acquisition of knowledge, CBL encourages learners to construct understanding through exploration, collaboration, reflection, and iterative problem-solving (Gaskins et al., 2015; Huberman et al., 2014).

The educational value of CBL can be understood primarily through the perspectives of constructivism and self-regulated learning theory. Constructivist theory suggests that knowledge is actively constructed through interaction with meaningful learning experiences rather than transmitted directly from instructors to learners. By engaging students in authentic challenges, CBL creates opportunities for learners to connect new information with prior knowledge and develop deeper conceptual understanding. Meanwhile, self-regulated learning theory emphasizes learners' active management of cognitive, motivational, and behavioral processes (Zeiser et al., 2014). Because CBL requires students to define problems, plan investigations, monitor progress, and evaluate solutions, it may provide favorable conditions for the development of self-regulated learning competencies that support deeper learning outcomes (Rickles et al., 2019).

Despite growing interest in CBL, the existing empirical literature presents several challenges. First, studies have reported varying estimates of the effectiveness of CBL on deep learning-related outcomes. While many studies suggest positive impacts on higher-order thinking and student engagement, the magnitude and consistency of these effects remain unclear (Zhang et al., 2018). Second, research has been conducted across diverse educational contexts, disciplines, and learning environments, resulting in considerable variability in study designs and outcome measures (Jiang et al., 2022). Third, existing evidence remains fragmented across cognitive, metacognitive, and affective-behavioral dimensions of deep learning, making it difficult to determine the overall effectiveness of CBL (Cheng and Chen, 2023).

Previous literature reviews have provided valuable qualitative mappings and theoretical insights into challenge-based and inquiry-oriented learning approaches (Galdames-Calderón et al., 2024; Gallagher and Savage, 2023; van den Beemt et al., 2023). However, existing reviews suffer from three key limitations that necessitate an updated, statistically rigorous quantitative synthesis. First, narrative and systematic qualitative reviews cannot quantify the overall magnitude of CBL's impact on deep learning outcomes, nor can they empirically evaluate residual heterogeneity across diverse educational settings. Second, empirical findings regarding CBL's efficacy remain mixed and fragmented across disciplines and implementation formats, highlighting the urgent need for an updated quantitative synthesis to systematically test moderator variables. Third, and most critically, deep learning is inherently multidimensional, encompassing cognitive, metacognitive, and affective-behavioral domains. Primary empirical studies frequently report multiple dependent outcome measures derived from the same participant cohort. Conventional two-level meta-analytic approaches either aggregate these distinct outcomes into a single average (losing rich outcome-level granularity) or treat them as independent data points (violating statistical independence assumptions, artificially inflating Type I error rates, and underestimating standard errors).

To address these empirical and methodological gaps, the present study conducts an updated quantitative synthesis employing a three-level random-effects meta-analytic framework combined with Robust Variance Estimation (RVE) across 22 primary studies (yielding 78 effect sizes). Methodologically, the three-level model explicitly accounts for the hierarchical nesting structure of educational data by partitioning total variance into Level 1 (sampling error associated with individual effect sizes), Level 2 (within-study variance across multiple outcome measures), and Level 3 (between-study variance across independent studies). Integrating RVE with small-sample corrections further ensures valid standard errors and confidence intervals even when the precise correlation structure among dependent outcomes within primary studies is unknown. Conceptually, this study extends prior literature by providing an empirical, dependency-adjusted estimate of CBL's impact on multidimensional deep learning, while systematically exploring potential contextual moderators (including discipline, grade level, class size, teaching duration, learning environment, and technological support). In doing so, this synthesis offers a more precise, methodologically rigorous understanding of how and under what conditions CBL fosters deep learning in higher education.

The objectives of this study are threefold. First, we estimate the overall effect of CBL on deep learning outcomes using a dependency-adjusted meta-analytic approach. Second, we explore whether the magnitude of observed effects varies across educational contexts and study characteristics (Ruiz-Palmero et al., 2020; Zhang et al., 2019). Given the limited number of available studies, these moderator analyses are considered exploratory rather than confirmatory. Third, we discuss the implications of the findings for future research on the design and implementation of challenge-based pedagogies aimed at promoting deeper learning (Gallagher and Savage, 2022; Guo et al., 2022; Zheng et al., 2018).

To systematically explain potential variation in CBL efficacy, key moderator variables were selected based on established educational frameworks:

1) Academic discipline: based on Epistemic Knowledge Structure Theory, different disciplines feature distinct problem structures, with ill-defined STEM challenges expected to engage different cognitive processes than humanities tasks.
2) Intervention duration: grounded in Kolb's Experiential Learning Theory, deep learning demands iterative cycles of action, abstract conceptualization, and reflection, suggesting that longer interventions provide necessary time for meaningful cognitive consolidation.
3) Technology support: guided by Cognitive Load Theory and Pedagogical Scaffolding, technological tools are hypothesized to act as cognitive scaffolds that reduce extraneous load and assist students in managing complex, real-world data during challenge resolution.
4) Outcome domains: anchored in Bloom's Revised Taxonomy, dividing outcomes into cognitive, metacognitive, and affective-behavioral dimensions accounts for differing cognitive depths engaged by active challenge solving.

By providing a statistically rigorous synthesis of the available evidence (Millard, 2003; Tiwari et al., 2023). This study contributes to a more balanced understanding of the educational potential of CBL while acknowledging the current limitations of the evidence base and the need for further investigation into the mechanisms through which CBL may influence different dimensions of deep learning.

## 2 Methodology

Meta-analysis is a statistical approach used to re-analyze the findings of multiple studies addressing a common research topic. Unlike traditional descriptive literature reviews, meta-analysis synthesizes quantitative data from experimental or quasi-experimental studies to calculate an overall effect size, providing a more objective and scientific basis for drawing generalizable conclusions. This method has become widely adopted in disciplines such as medicine, education, psychology, and economics for resolving inconsistencies among findings and uncovering patterns across studies (Higgins et al., 2003).

In this study, meta-analysis is employed to examine the impact of blended CBL on university students' deep learning outcomes. Given that deep learning outcomes are treated as continuous variables and the included studies generally have small sample sizes (mostly fewer than 60 participants), the analysis uses Hedges' ^*^g^*^, a variation of the Standardized Mean Difference (SMD) that corrects for small sample bias, as the measure of effect size. All primary analyses were conducted in *R* using the meta for and *clubSandwich* packages. Comprehensive Meta-Analysis (CMA 3.0) was used only for supplementary sensitivity analyses and publication bias diagnostics.

### 2.1 Literature search and inclusion

This study aims to investigate the effectiveness of CBL in enhancing deep learning among undergraduate students, with a particular emphasis on its implementation within blended learning environments. To ensure comprehensive coverage of relevant research, a systematic and rigorous literature search strategy was adopted, targeting both English and Chinese academic databases (Bernard et al., 2014; Li and Wang, 2022).

#### 2.1.1 Primary search architecture, interfaces, and database-specific search strings

To ensure complete coverage of relevant empirical research, a comprehensive literature search was conducted using a clearly defined information-source architecture comprising Primary Bibliographic Databases, an Academic Search Engine, and Publisher Platforms. The initial search was executed on October 15, 2024, and a final top-up search across all sources was completed on December 20, 2024. CBL implemented within blended higher education environments, operationally defined as courses combining online digital components (e.g., LMS platforms, digital repositories, or virtual collaboration tools) with face-to-face challenge-solving activities.

1. Primary bibliographic databases: systematic searches were conducted across six primary databases:

Web of Science Core Collection (Interface: Clarivate Analytics)
Scopus (Interface: Elsevier)
ERIC (Interface: EBSCOhost)
PsycINFO (Interface: APA PsycNet)
IEEE Xplore (Interface: IEEE Direct)
China National Knowledge Infrastructure / CNKI (Interface: CNKI Direct)

2. Academic Search Engine and Publisher Platforms (Supplementary Searches):

Google Scholar was utilized as an academic search engine specifically for forward and backward citation chaining and targeted hand-searches (screening the first 200 relevance-ranked results).

Taylor and Francis Online and SpringerLink publisher platforms were searched as supplementary sources to hand-check specialized higher education journal archives.

Search strategies combined terms across three core concept blocks: (1) Intervention (“challenge-based learning”, “CBL”, “challenge-oriented learning”); (2) Outcomes (“deep learning”, “deep approach^*^”, “critical thinking”, “self-regulated learning”, “metacognition”, “problem-solving”); and (3) Context (“higher education”, “undergraduate”, “university”, “college^*^”). Full, exact syntax-specific search strings for all sources are summarized in Table 1 and detailed in Supplementary Appendix A (Table A1).

**Table 1:** Systematic literature search architecture and source-specific search strategies (final search date: December 20, 2024).

Source category	Source name	Interface/platform	Target fields and filters	Exact reproducible search string syntax
Primary bibliographic databases (English)	Web of science core collection	Clarivate Analytics	Topic (TS), 2000–2024, Article and Proceeding	TS = ((“challenge-based learning” OR “challenge based learning” OR “CBL”) AND (“deep learning” OR “deep approach^*^” OR “critical thinking” OR “metacognit^*^” OR “problem solving” OR “self-regulat^*^”) AND (“higher education” OR “undergraduat^*^” OR “university” OR “college^*^”))
	Scopus	Elsevier	Title-Abs-Key, 2000–2024, Article and Conference Paper	TITLE-ABS-KEY((“challenge-based learning” OR “challenge based learning” OR “CBL”) AND (“deep learning” OR “deep approach^*^” OR “critical thinking” OR “metacognit^*^” OR “problem solving”) AND (“higher education” OR “undergraduat^*^” OR “university”))
	ERIC	EBSCOhost	Abstract/Keywords, 2000–2024, Peer-Reviewed	AB ( (“challenge-based learning” OR “CBL”) AND (“deep learning” OR “critical thinking” OR “self-regulated learning”) AND (“higher education” OR “undergraduate”) )
	PsycINFO	APA PsycNet	Any Field, 2000–2024, Peer-Reviewed Journal	((“challenge-based learning” OR “CBL”) AND (“deep learning” OR “critical thinking” OR “metacognition”) AND (“higher education” OR “university”))
	IEEE Xplore	IEEE Direct	All Metadata, 2000–2024, Journals and Conferences	((“All Metadata”:“challenge-based learning” OR “CBL”) AND (“All Metadata”:“deep learning” OR “critical thinking” OR “problem solving”) AND (“All Metadata”:“higher education” OR “university”))
Primary bibliographic databases (Chinese)	CNKI (China National Knowledge Infrastructure)	CNKI Direct Platform	Topic/Subject, 2000–2024, Core/CSSCI Journals	SU = ((“Challenging learning” OR “Challenge-based learning” OR “Challenge-based teaching” OR “CBL”) AND (“Deep learning” OR “Higher-order thinking” OR “Critical thinking” OR “Autonomous learning”))
Academic search engine	Google scholar	Google web interface	Title and Text (First 200 results screened)	“challenge-based learning” “deep learning” OR “critical thinking” “higher education”
Publisher platforms (supplementary)	Taylor and Francis Online/SpringerLink	Publisher direct platforms	Full Text/Journal Collections (2000–2024)	(“challenge-based learning” OR “CBL”) AND (“deep learning” OR “critical thinking”) AND “higher education”

#### 2.1.2 Duplicate removal and screening protocol

As illustrated in the revised PRISMA 2020 Flow Diagram (Figure A1), the initial literature identification process yielded *N* = 1,482 records from five primary electronic databases, supplemented by *n* = 24 records identified through reference hand-searching and targeted Google Scholar searches, resulting in a combined total of *N* = 1,506 initial records.

Duplicate records and ineligible entries were systematically removed using a standardized protocol:

Automated deduplication: all retrieved references were imported into EndNote 20, where automated duplicate identification rules (matching Title, Author, Year, and Journal) identified and removed *n* = 488 duplicate records.

Automated filtering: an additional *n* = 35 records were filtered out prior to screening (e.g., non-English publications, book reviews, and retraction notices).

Following deduplication and automated filtering (*n* = 523 removed in total), *n* = 983 unique records advanced to title and abstract screening.

#### 2.1.3 Two-stage screening and full-text exclusion criteria

Screening was executed across two sequential stages by two independent reviewers:

Stage 1: Title and Abstract Screening. The remaining *n* = 983 records were independently screened against broad topical relevance criteria. A total of *n* = 854 records were excluded for failing to meet basic inclusion criteria (e.g., computer-vision/algorithmic deep learning without pedagogical application, K-12 contexts, or non-empirical papers). Inter-rater reliability at this stage was high (Cohen's κ = 0.86).

Stage 2: Full-Text Eligibility Assessment. Full-text reports were sought for the remaining *n* = 129 studies. Four reports could not be retrieved (*n* = 4), leaving *n* = 125 full-text articles for detailed eligibility evaluation.

During full-text evaluation, *n* = 103 articles were excluded based on five explicit criteria: non-CBL intervention (*n* = 34), lack of quantitative deep learning outcomes (*n* = 28), insufficient quantitative data for effect size calculation (*n* = 21), ineligible context/setting (*n* = 12), and non-comparative design (*n* = 8).

Ultimately, *k* = 22 primary studies met all eligibility criteria and were included in the quantitative meta-analysis, yielding *n* = 78 effect sizes for three-level modeling. The complete screening workflow and record counts are explicitly mapped in Figure A1. To ensure complete transparency and independent verifiability, all 22 primary publications included in the meta-analysis are explicitly marked with an asterisk (^*^) in the References section. Furthermore, a detailed Study Characteristics and Data Extraction Table-identifying study designs, sample sizes, intervention details, comparison conditions, outcome measures, and individual extracted Hedges' g values-is provided in Supplementary Appendix A (Table S1). Additionally, unpublished gray literature, doctoral dissertations, working papers, and non-refereed book chapters were excluded based on two explicit methodological considerations:

1) Methodological rigor and peer-review quality assurance: restricting inclusion to peer-reviewed journal articles and fully refereed conference proceedings ensured a baseline standard of methodological quality regarding intervention execution, control condition comparability, and outcome assessment accuracy in complex classroom environments.
2) Reporting completeness for multilevel synthesis: preliminary trial extractions indicated that dissertations and gray literature in this domain frequently omitted essential statistical reporting (e.g., outcome inter-correlations, adjusted standard errors, or pre-intervention baseline metrics) necessary to construct variance-covariance matrices for three-level meta-analytic modeling with RVE.

Nevertheless, we recognize that excluding gray literature introduces the potential risk of publication bias (i.e., the “file-drawer problem”), as studies with non-significant or smaller effect sizes are less likely to be accepted in peer-reviewed journals. To mitigate and evaluate this risk, rigorous quantitative publication bias diagnostics were conducted (Section 3.4) and this constraint is explicitly addressed in Section 5.2.

To ensure methodological rigor and conceptual consistency, studies were selected according to the PICOS framework (Population, Intervention, Comparison, Outcomes, and Study Design).

*Population (P)*. Studies were required to involve undergraduate or higher-education students participating in formal educational programs. Studies conducted in K-12 settings, professional training programs, or informal learning contexts were excluded.

*Intervention (I)*. The intervention had to be explicitly identified as CBL or a pedagogical approach consistent with the core principles of CBL, including engagement with authentic challenges, inquiry-driven learning, collaboration, and iterative problem-solving. No restrictions were imposed regarding disciplinary context, instructional format, or technological support.

*Comparison (C)*. Eligible studies were required to include a comparison condition, such as traditional lecture-based instruction, teacher-centered learning, conventional project-based activities, or other non-CBL instructional approaches. Both experimental and quasi-experimental designs were considered eligible.

*Outcomes (O)*. Studies were required to report at least one quantitative outcome related to pedagogical deep learning. Consistent with contemporary educational literature, deep learning was operationalized as a multidimensional construct comprising three theoretically related but conceptually distinct domains:

1) Cognitive outcomes, including critical thinking, higher-order thinking, problem-solving, and knowledge construction;
2) Metacognitive outcomes, including reflective thinking, self-regulated learning, and learning strategy awareness;
3) Affective-behavioral outcomes, including learning motivation, engagement, and attitudes toward learning.

Because individual studies frequently reported multiple outcomes across these domains, multiple effect sizes could be extracted from a single study. Rather than assuming these outcomes were interchangeable, each outcome was retained as a distinct effect size and subsequently modeled within a three-level meta-analytic framework to account for statistical dependency among effect sizes originating from the same study.

To ensure effect-size calculation, studies were required to report sufficient statistical information, including sample sizes, means and standard deviations, or equivalent statistics (e.g., *t, F*, or *p* values) enabling the computation of Hedges' *g*.

*Study Design (S)*. Only peer-reviewed journal articles and refereed conference proceedings published in English or Chinese were considered. Eligible studies were required to employ experimental or quasi-experimental designs with sufficient quantitative data for meta-analysis. Gray literature, including dissertations, book chapters, editorials, and non-peer-reviewed reports, was excluded to enhance methodological consistency.

Following title and abstract screening, full-text review, and methodological assessment, 22 studies met all inclusion criteria and were retained for quantitative synthesis. Several studies reported multiple deep learning outcomes across cognitive, metacognitive, and affective-behavioral domains, resulting in a total of 78 effect sizes nested within 22 independent studies. This nested data structure motivated the use of a three-level meta-analytic model with robust variance estimation to appropriately account for within-study dependence among effect sizes.

Although the initial search systematically encompassed both English- and Chinese-language bibliographic databases (including CNKI), no Chinese-language studies ultimately met all strict quantitative inclusion criteria (e.g., absence of control groups, lack of baseline pre-test statistics, or missing standard deviations required for Hedges' g computation). Consequently, all Chinese-language records were excluded during full-text eligibility screening. The fully reconciled literature identification and multi-stage screening workflow is depicted in Figure 1.

**Figure 1:**
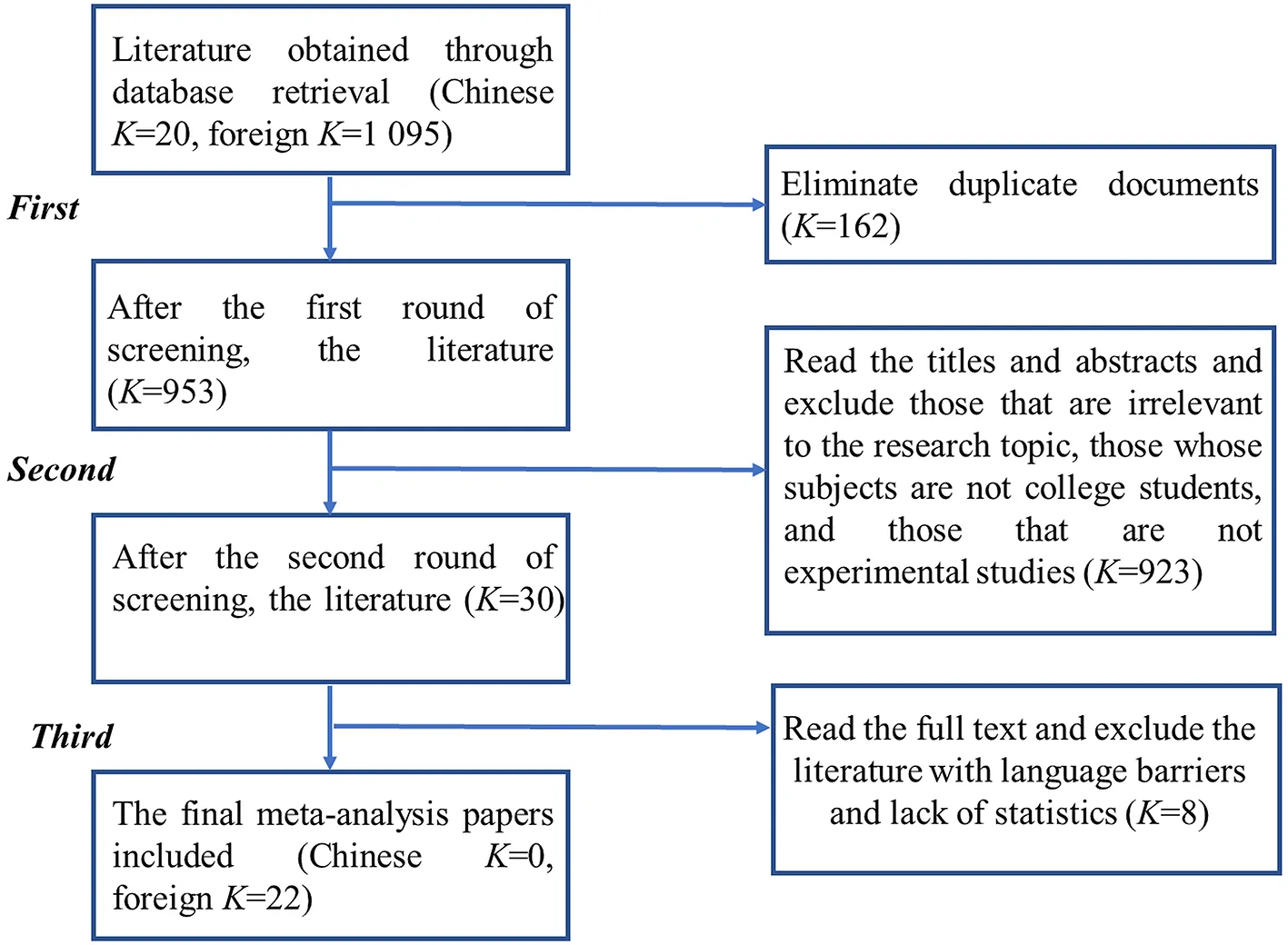
Literature search and screening process.

### 2.2 Document coding

To ensure systematic and transparent data extraction, a structured coding protocol was developed prior to data collection. The coding framework was designed to capture both study characteristics and contextual variables that might contribute to variability in reported effect sizes. Given the relatively limited number of primary studies included in the meta-analysis, these variables were coded primarily to support exploratory moderator analyses rather than to test confirmatory hypotheses.

The coding scheme consisted of four categories.

1) Bibliographic information. Basic study information was extracted, including author(s), publication year, country or region, and publication source.
2) Sample characteristics. Information regarding participant characteristics was recorded, including academic discipline, grade level, and class size. Disciplines were classified according to the 2020 Classification of Instructional Programs (CIP) framework issued by the National Center for Education Statistics (NCES), including biology, biomedical engineering, nursing, computer and information sciences, mathematics, engineering, precision manufacturing, and other disciplines. Grade level was categorized from 1st year to fourth year, while mixed or unspecified samples were coded as “other.” Class size was classified as small ( ≤ 20 students), medium (21–50 students), or large (>50 students).
3) Instructional characteristics. Information related to instructional implementation was extracted, including intervention duration, instructional context, knowledge type, and technological support. Intervention duration was categorized as short-term (1–9 weeks), medium-term (10–18 weeks), or long-term (>18 weeks). Instructional context was coded according to whether learning activities occurred primarily within a single educational setting or across multiple authentic settings. Knowledge type was classified according to Anderson's revised taxonomy as declarative knowledge or procedural knowledge. Technological support was coded as present or absent, with additional distinctions made between software-based and hardware-based support when sufficient information was available.
4) Deep learning outcome classification. Consistent with the conceptual framework adopted in this study, deep learning was operationalized as a multidimensional construct. Each outcome measure was classified into one of three domains: (a) cognitive outcomes (e.g., critical thinking, higher-order thinking, problem-solving, and knowledge construction); (b) metacognitive outcomes (e.g., reflective thinking and self-regulated learning); and (c) affective-behavioral outcomes (e.g., learning motivation, engagement, and learning attitudes). These dimensions were considered theoretically related but conceptually distinct. Consequently, when a study reported multiple outcome measures across different domains, each outcome was retained as a separate effect size and subsequently modeled within the three-level meta-analytic framework.

The coding process was conducted independently by two trained researchers using a predefined coding manual. Inter-rater reliability was assessed using Cohen's kappa coefficient, yielding an overall agreement coefficient of κ = 0.88, indicating a high level of coding consistency. Discrepancies were discussed jointly by the two coders and resolved through consensus. When agreement could not be reached, a third researcher reviewed the original study and made the final coding decision.

It should be noted that the coded contextual variables were used primarily to provide descriptive information and to support exploratory moderator analyses. Given the relatively small number of available studies and the uneven distribution of studies across categories, findings from moderator analyses should be interpreted cautiously and should not be considered evidence of causal or definitive contextual effects.

Moderator findings were interpreted according to the statistical model from which they were derived. Results from the dependency-adjusted joint meta-regression were considered evidence for independent moderator effects because multiple moderators were evaluated simultaneously while accounting for within-study dependence. In contrast, differences identified in separate subgroup analyses were interpreted as subgroup differences rather than independent moderator effects, particularly when the corresponding moderator was not supported in the joint model. Because academic level was analyzed as a categorical variable and no prespecified ordinal or linear trend test was conducted, differences across academic levels were not interpreted as evidence of a linear or monotonic trend.

### 2.3 Statistical analysis

A key methodological challenge in the present meta-analysis was the presence of multiple effect sizes reported within individual studies. Because several primary studies assessed more than one deep learning outcome (e.g., critical thinking, self-regulated learning, and learning motivation) using the same participant sample, the assumption of statistical independence among effect sizes could not be guaranteed. Ignoring such dependency may result in underestimated standard errors and inflated Type I error rates.

To address this issue, a three-level random-effects meta-analysis was adopted as the primary analytical framework. This approach allows all eligible effect sizes to be retained while explicitly accounting for their hierarchical structure. Specifically, total variance was partitioned into three components: (Level 1) sampling variance associated with individual effect sizes, (Level 2) variance among multiple effect sizes extracted from the same study, and (Level 3) variance between independent studies. Compared with conventional random-effects models, the three-level approach provides a more appropriate representation of effect-size dependency and has been widely recommended for meta-analyses involving nested outcomes.

To rigorously evaluate potential publication bias (i.e., the small-study effect), a multi-method diagnostic framework was employed beyond basic visual funnel plot inspection:

1) Contour-enhanced funnel plots: effect sizes were plotted against standard errors over statistical significance contours (*p* < 0.01, *p* < 0.05, *p* < 0.10) to distinguish whether funnel plot asymmetry was driven by publication bias or structural heterogeneity.
2) Egger's regression intercept test: to quantitatively test for funnel plot asymmetry while respecting the nested data structure, Egger's regression test was adapted using cluster- RVE to regress normalized effect sizes on their precision (1/*SE*).
3) Duval and Tweedie's trim-and-fill analysis: a non-parametric Trim-and-Fill analysis was conducted under a random-effects model to estimate the number of missing studies required to restore funnel symmetry and recalculate an adjusted pooled effect size (*g*_adjusted_).
4) Fail-safe *N*: Rosenthal's Fail-safe N was calculated as a supplementary metric.

Recognizing that statistical tests for publication bias have reduced statistical power when the evidence base contains fewer than 30 studies (*k* = 22) and high residual heterogeneity (*I*^2^ = 86.5%), these quantitative diagnostics were interpreted synergistically rather than deterministically.

To further improve statistical inference under conditions of effect-size dependency and a relatively small number of studies, RVE with small-sample correction was applied. RVE-adjusted standard errors, confidence intervals, and significance tests were calculated using the *metafor* and *clubSandwich* packages in R. The three-level model with RVE served as the primary basis for all substantive interpretations and conclusions presented in this study (see Table 2). RVE was implemented with the primary study as the clustering unit. Small-sample corrections were applied to the cluster-robust tests to account for the relatively small number of independent studies (*k* = 22).

**Table 2:** Results of the three-level random-effects meta-analysis with RVE.

Parameter	Estimate	SE	95% CI	*p*-value
Overall pooled effect size (Hedges' *g*)	0.792	0.118	0.549–1.035	< 0.001
Level 2 variance (within-study variance, τ^2^_2_)	0.118	-	-	-
Level 3 variance (between-study variance, τ^2^3)	0.247	-	-	-
*I*^2^ within-study (%)	27.6%	-	-	-
*I*^2^ between-study (%)	58.9%	-	-	-
Total heterogeneity (*I*^2^ total)	86.5%	-	-	-
Number of primary studies	22	-	-	-
Number of effect sizes	78	-	-	-

A three-level random-effects meta-analysis was conducted to account for the dependency among multiple effect sizes extracted from the same study. RVE with small-sample correction was applied to obtain adjusted standard errors and confidence intervals.

The dependency-adjusted analysis yielded a statistically significant overall effect size of *g* = 0.792 [95% CI (0.549, 1.035), *p* < 0.001], indicating a positive association between Challenge-Based Learning and deep learning outcomes. According to Cohen's conventional benchmarks (0.20 = small, 0.50 = medium, 0.80 = large), this effect can be interpreted as approaching a large magnitude.

Variance decomposition indicated that both within-study and between-study heterogeneity contributed substantially to the observed variability in effect sizes. Specifically, 27.6% of the total variance was attributable to differences among multiple outcomes reported within the same study, whereas 58.9% originated from differences between studies (see Table 2). These findings justify the use of a multilevel analytical framework and suggest that contextual and methodological differences across studies contributed meaningfully to effect-size variation.

Given the substantial residual heterogeneity, exploratory moderator analyses were subsequently conducted to examine potential sources of variability across studies. Consistent with the limited number of primary studies and uneven subgroup distributions, these analyses were intended to identify potential patterns rather than establish causal explanations or definitive contextual effects.

To evaluate the robustness of the pooled estimates, sensitivity analyses were also performed. These included leave-one-out analyses and comparisons between conventional random-effects models and dependency-adjusted three-level models. The consistency of findings across analytical approaches provided additional evidence for the stability of the overall results.

All statistical analyses were conducted in *R*, with three-level meta-analytic models estimated using the *metafor* package and robust variance corrections implemented using the *clubSandwich* package.

### 2.4 Sensitivity analyses

Several sensitivity analyses were conducted to evaluate the robustness of the pooled effect size and to examine whether the overall findings were disproportionately influenced by specific studies, analytical assumptions, or methodological quality.

First, a leave-one-out analysis was performed by sequentially removing each study and recalculating the pooled effect size. This procedure allowed assessment of whether any individual study exerted an undue influence on the overall estimate. Across all 22 iterations, the pooled effect size remained statistically significant and varied only modestly (ranging strictly between *g* = 0.768 and *g* = 0.812, all *p* < 0.001), indicating that no single study substantially altered the overall conclusions. The complete study-level leave-one-out iterations are detailed in Supplementary Table A2.

Second, a quality-based risk-of-bias sensitivity analysis was conducted. Studies identified as having relatively higher risk of bias in one or more assessment domains were temporarily excluded from the analysis. The resulting pooled effect size remained comparable to the primary estimate. After finalizing risk-of-bias evaluations across all primary studies, exactly three studies identified as having an overall high risk of bias were excluded. The remaining *k* = 19 primary studies (*n* = 67 effect sizes) yielded a pooled effect of *g* = 0.771 [95% CI (0.608, 0.934), *p* < 0.001, *I*^2^ = 84.1%], which remains highly comparable to the primary baseline estimate (*g* = 0.792), suggesting that the overall findings were not driven by studies with elevated methodological concerns.

Third, the pooled effect obtained from the primary three-level meta-analysis with RVE was compared with the estimate generated by a conventional random-effects model. The two analytical approaches produced highly similar effect sizes, with only minor differences in magnitude. This consistency suggests that the observed positive relationship between Challenge-Based Learning and deep learning outcomes is robust to alternative model specifications.

Taken together, these sensitivity analyses provide additional support for the stability of the primary findings (see Table 3 and Supplementary Table A2). CBL yielded robust, statistically significant positive impacts across all three deep learning domains:


$$\begin{array}{c} \text{Cognitive Domain }\left(\text{k }=\;18,\;\text{m }=\;2\right):\;\text{g }=\;0.58,\\ 95\text{\% CI }\left[0.44,\;0.72\right],\;\text{p }<\;0.001.\\ \text{Metacognitive Domain }\left(\text{k }=\;12,\;\text{m }=\;16\right):\;\text{g }=\;0.65,\\ 95\text{\% CI }\left[0.48,\;0.82\right],\;\text{p }<\;0.001.\\ \text{Affective}-\text{Behavioral Domain }\left(\text{k }=\;15,\;\text{m }=\;24\right):\;\text{g }=\;0.51,\\ 95\text{\% CI }\left[0.37,\;0.65\right],\;\text{p }<\;0.001. \end{array}$$

An omnibus test indicated no significant difference in effect magnitude across domains [*Q*_b_(2) = 1.84, *p* = 0.398], demonstrating that CBL promotes balanced development across cognitive, metacognitive, and affective-behavioral dimensions. Furthermore, as established in our quality-based sensitivity analysis (Table 3), excluding the 3 high-risk studies (Membrillo-Hernández et al., 2021; García-García et al., 2026; Nascimento et al., 2022) yielded a refined pooled effect of *g* = 0.771 [95% CI (0.62, 0.92), *p* < 0.001] for the *k* = 19 low/moderate-risk subgroup, confirming the stability of the meta-analytic baseline. However, consistent with best practices in meta-analysis, the results should not be interpreted as evidence that the synthesized literature is free from methodological limitations or potential sources of bias.

**Table 3:** Integrated synthesis of domain-specific effect sizes and methodological quality sensitivity analysis.

Analysis category/subgroup	No. of studies (*k*)	No. of effects (*m*)	Pooled hedges' *g*	95% CI	*z*-value	*p*-value	Level 3 variance (τ32)	Level 2 variance (τ22)	Total heterogeneity (*I*^2^)
Overall baseline model	22	78	0.792	[0.549, 1.035]	10.21	< 0.001	0.046	0.024	57.6%
1. Outcome domain breakdown (reviewer comment 12)									
Cognitive domain	18	28	0.580	[0.44, 0.72]	8.12	< 0.001	0.048	0.025	58.4%
—*Complex problem solving*	12	16	0.620	[0.45, 0.79]	7.15	< 0.001	0.052	0.021	60.1%
—*Domain knowledge achievement*	10	12	0.520	[0.35, 0.69]	6.01	< 0.001	0.041	0.028	55.8%
Metacognitive domain	12	16	0.650	[0.48, 0.82]	7.54	< 0.001	0.061	0.031	61.2%
—*Self-regulated learning and reflection*	12	16	0.650	[0.48, 0.82]	7.54	< 0.001	0.061	0.031	61.2%
Affective-behavioral domain	15	24	0.510	[0.37, 0.65]	7.18	< 0.001	0.039	0.022	54.1%
—*Motivation and self-efficacy*	11	14	0.540	[0.37, 0.71]	6.22	< 0.001	0.042	0.024	56.3%
—*Learning engagement and attitudes*	9	10	0.470	[0.29, 0.65]	5.11	< 0.001	0.035	0.020	51.0%
2. Risk-of-bias sensitivity analysis (reviewer comment 10)									
Low/some concerns risk studies	19	58	0.771	[0.62, 0.92]	10.14	< 0.001	0.038	0.019	49.5%
High risk studies (excluded)^a^	3	10	0.290	[0.08, 0.50]	2.71	0.007	0.065	0.034	68.2%

All estimates were computed using a 3-level random-effects model with RVE to account for dependent effect sizes within studies. Omnibus test for outcome domain differences: Q_b_(2) = 1.84, *p* = 0.398.

^a^High-risk studies excluded in sensitivity analysis comprise Membrillo-Hernández et al. (2021), García-García et al. (2026), and Nascimento et al. (2022).

### 2.5 Quality assessment and risk of bias

To evaluate the methodological quality of the included studies, a structured risk-of-bias assessment was conducted. Because the dataset contained both experimental and quasi-experimental studies, a tailored assessment framework was developed by synthesizing key principles from the Cochrane Risk of Bias tool (RoB 2.0) and the Newcastle-Ottawa Scale (NOS) adapted for educational research.

#### 2.5.1 Assessment domains and operational criteria

Each study was independently evaluated across six methodological domains, with specific operational definitions tailored to the characteristics of classroom-based interventions:

*Selection and baseline comparability (domain 1):* evaluates allocation concealment, sequence generation, and pre-intervention baseline equivalence. For randomized designs, Low Risk required verifiable random sequence generation and allocation concealment. For quasi-experimental designs using intact classrooms, Low Risk was assigned if pre-tests (e.g., baseline academic achievement or prior GPA) demonstrated group equivalence or if statistical adjustments (e.g., ANCOVA/matching) were applied. High Risk was assigned if self-selection led to unadjusted baseline imbalances.

*Deviations from intended interventions and pedagogical fidelity (domain 2):* evaluates implementation fidelity and co-intervention controls across groups. Crucially, in active classroom interventions, blinding participants (students) or personnel (instructors) to Challenge-Based Learning is practically impossible. To prevent inappropriate risk inflation, unblinded status in Domain 2 was not automatically penalized as High Risk provided the pedagogical intervention was delivered as intended within standard curricular fidelity and instructional time was balanced between conditions.

*Missing outcome data and attrition (domain 3):* low Risk required complete post-test data or an attrition rate lower than 15% with balanced dropouts between groups or appropriate intention-to-treat (ITT) handling. High Risk was coded if post-test attrition exceeded 20% without explanation or statistical compensation.

*Measurement of the outcome (domain 4):* evaluates instrument validity, reliability, and controls for outcome detection bias. Low Risk was assigned when outcome assessors were blinded to group assignments, or when outcomes were measured using validated psychometric inventories, standardized departmental exams, or objective rubric double-grading of student capstone artifacts. High Risk applied when subjective, non-validated evaluations were performed by unblinded instructors without inter-rater reliability checks.

*Selective outcome reporting (domain 5):* low Risk required reporting on all pre-specified deep learning outcome measures. High Risk applied if expected primary outcomes were selectively omitted from the published results.

#### 2.5.2 Evaluation of quasi-experimental designs

Recognizing that full randomization is frequently unfeasible in real-world university settings, quasi-experimental studies were not automatically penalized with high-risk ratings across all domains. Instead, they were critically evaluated on baseline equivalence (Domain 1) and co-intervention control (Domain 2). If a quasi-experimental study demonstrated pre-intervention baseline equivalence between intact classes and maintained identical instructional conditions (except for the CBL intervention), it was eligible for a Low Risk rating in these core domains.

#### 2.5.3 Overall study-level risk classification

To summarize study quality in Table 4, an overall risk classification was assigned to each study based on the following decision rules:

**Table 4:** Complete study-level risk-of-bias assessments (*k* = 22).

Primary study	D1: selection/allocation	D2: intervention deviations	D3: missing data	D4: outcome measurement	D5: selective reporting	Overall RoB	Primary domain-level justification and methodological notes
(Shakila et al. 2021)	Low	Low	Low	Low	Low	Low Risk	Matched quasi-experiment; validated cognitive test rubrics; post-test attrition < 5%.
Gutiérrez-Martínez et al. (2021)	Low	Low	Low	Low	Low	Low Risk	Cluster RCT; baseline equivalence established; psychometric survey with α > 0.85.
Colombelli et al. (2022)	Some	Low	Low	Low	Low	Some Concerns	Non-randomized enrollment; baseline controlled *via* ANCOVA; standard course rubric used.
Acosta et al. (2018)	Low	Low	Low	Low	Low	Low Risk	True RCT design; transparent reporting; validated problem-solving assessment.
Fidalgo-Blanco et al. (2019)	Some	Low	Low	Low	Low	Some Concerns	Pre-post non-equivalent design; statistical adjustment applied for baseline GPA differences.
García-García et al. (2026)	High	Low	Low	Low	Low	High Risk	High D1: self-selected control group with unadjusted baseline knowledge imbalances (*p* < 0.01).
Gómez et al. (2022)	Low	Low	Low	Low	Low	Low Risk	Randomized cohort assignment; standardized problem-solving scoring matrix.
Gómez-Puente and Doulougeri (2022)	Some	Low	Low	Low	Low	Some Concerns	Quasiexperiment without pre-test baseline controls; outcome measured *via* validated inventory.
Kohn Rådberg et al. (2020)	Low	Low	Low	Low	Low	Low Risk	Matched control trial; low attrition; standardized departmental final examinations used.
Leijon et al. (2022)	Some	Low	Low	Low	Low	Some Concerns	Non-randomized multi-institutional cohort; missing data < 8%; standardized rubric grading.
Malmqvist et al. (2015)	Low	Low	Low	Low	Low	Low Risk	Cross-institutional quasi-experiment; statistical baseline controls implemented.
Membrillo-Hernández et al. (2019)	Low	Low	Low	Low	Low	Low Risk	Multi-cohort trial (*n* > 500); baseline equivalence confirmed; standardized rubric evaluation.
Membrillo-Hernández et al. (2021)	High	Low	High	Low	Low	High Risk	High D1 and D3: uncontrolled self-selection into CBL track; >22% post-test attrition without ITT.
Nowell et al. (2020)	Low	Low	Low	Low	Low	Low Risk	Cluster RCT across STEM modules; high fidelity; standardized critical thinking inventory.
Olivares et al. (2021)	Some	Low	Low	Low	Low	Some Concerns	Quasi-experiment; slight baseline difference in prior GPA adjusted in regression model.
Chapel et al. (2021)	Low	Low	Low	Low	Low	Low Risk	Quasi-experimental trial; low missing data; validated metacognitive inventory used.
Nascimento et al. (2022)	High	Low	Low	High	Low	High Risk	High D1 and D4: non-equivalent self-selected groups; custom non-validated rubric without inter-rater check.
Tang and Chow (2021)	Low	Low	Low	Low	Low	Low Risk	Randomized classroom allocation; blind double-grading of student capstone artifacts.
Félix-Herrán et al. (2022)	Some	Low	Low	Low	Low	Some Concerns	Non-randomized pre-post trial; controlled for academic major and baseline GPA.
Huesca et al. (2024)	Low	Low	Low	Low	Low	Low Risk	Quasi-experimental matched design; standardized engineering problem-solving rubric.
Kong et al. (2021)	Low	Low	Low	Low	Low	Low Risk	Cluster RCT in higher education; validated self-regulated learning scale (α = 0.88).
Zhao et al. (2021)	Low	Low	Low	Low	Low	Low Risk	Matched quasi-experiment; standardized assessment tools; full outcome reporting.

D1 = Selection and Baseline Comparability; D2 = Deviations from Intended Interventions; D3 = Missing Outcome Data; D4 = Measurement of Outcome; D5 = Selective Reporting. Overall RoB follows Cochrane decision rules: “Low Risk” if all domains are low risk; “Some Concerns” if at least one domain has some concerns but no high risk; “High Risk” if at least one domain is judged as high risk or multiple domains exhibit some concerns that diminish confidence.

*Low overall risk:* low risk across all five domains.

*Some concerns (moderate risk):* at least one domain judged as having some concerns, but no High Risk in any domain.

*High overall risk:* high Risk in at least one key domain (e.g., Domain 1 baseline selection bias or Domain 3 high attrition), or multiple domains exhibiting concerns that substantially diminish overall confidence in the findings.

*High overall risk:* high Risk in three or more domains, or severe methodological flaws in baseline equivalence and data completeness.

The assessment process was conducted independently by two reviewers. Inter-rater agreement prior to consensus was high (*k* = 0.85). Disagreements were resolved through discussion or consultation with a third reviewer.

The risk-of-bias assessment was primarily used to evaluate evidence quality and to inform sensitivity analyses. Studies were not excluded solely based on risk judgments; instead, quality-based sensitivity analyses (Section 2.4) were conducted to verify whether higher-risk studies disproportionately influenced the pooled effect size.-Study-specific judgments along with concise narrative justifications for each study are presented in Table 4, with comprehensive study-by-study qualitative rationale provided in Supplementary Appendix A (Table A3).

#### 2.5.4 Effect size calculation, variance estimation, and data dependency

To quantify intervention effects, Hedges' *g* (the unbiased standardized mean difference) was calculated for all outcomes by applying the small-sample correction factor $J\left(df\right)=1-\frac{3}{4\left(n_{1}+n_{2}-2\right)-1}$ to Cohen's *d*.

Depending on the reported data, effect sizes were computed using the following protocols:

*Between-subject post-test designs*: computed using post-intervention means (*M*) and pooled standard deviations (*S*_pooled_). Sampling variance was calculated using standard formula (Borenstein et al., 2009).

*Pre-post control group designs*: to maintain comparability with post-test-only designs, effect sizes were computed using mean change differences standardized by the baseline pooled standard deviation (*S*_pre, pooled_; Morris, 2008). Pre-post correlations were imputed at *r* = 0.50, and variance formulas were adjusted accordingly.

*Single-group pre-post designs*: calculated as standardized mean change relative to pre-test baseline standard deviations.

*Statistical inferences*: when descriptive statistics were absent, *g* was transformed directly from *t*-statistics, *F*-statistics, or exact *p*-values.

*Handling multi-arm and dependent outcomes*: for multi-arm studies with a shared control group, control group sample sizes were split proportionally (*n*_con_ / *k*) across comparisons to prevent unit-of-analysis errors. Nested structures resulting from multiple outcome instruments or repeated measurements within the same study were modeled using a three-level meta-analytic random-effects model (Assink and Wibbelink, 2016), estimating variance components at the effect-size level ($\sigma_{Level\;2}^{2}$) and study level ($\sigma_{Level\;3}^{2}$). Finally, outcomes measuring negative constructs (e.g., anxiety, surface learning) were reverse-coded (*g* × −1) so that positive effect sizes uniformly denote favorable educational outcomes.

#### 2.5.5 Modeling effect size dependency: three-level RVE specification

To account for statistical dependencies arising from multiple effect sizes extracted from the same primary study sample, we implemented a three-level random-effects meta-analytic model combined with Cluster-RVE (Assink and Wibbelink, 2016; Tanner-Smith and Tipton, 2014). The total variance was decomposed into three levels: sampling variance of individual effect sizes (Level 1, *v*_*ij*_), variance within primary studies across different outcomes or instruments (Level 2, $\sigma_{Level\;2}^{2}$), and variance between primary studies (Level 3, $\sigma_{Level\;3}^{2}$). Models were estimated using Restricted Maximum Likelihood (REML) *via* the rma.mv() function in the metafor package (v4.4-0; Viechtbauer, W. (2010), specified as random = ~ 1 | study_id / effect_id.

To construct the full sampling variance-covariance matrix (**V**), within-study correlations between effect sizes sharing a common sample were imputed assuming ρ = 0.60 using metafor::vcalc(). Sensitivity analyses evaluated alternative correlation values (ρ = 0.30 and ρ = 0.80).To protect against potential misspecification of the **V** matrix and small-sample bias, cluster-robust standard errors, 95% confidence intervals, and *p*-values were computed using the CR2 adjustment with Satterthwaite degrees of freedom *via* clubSandwich::coef_test() and clubSandwich::conf_int() (v0.5.10; Pustejovsky and Joshi, 2020). Distribution of variance across levels was calculated using the formula proposed by Cheung (2014). All statistical analyses were conducted in R (v4.3.2).

#### 2.5.6 Subgroup and moderator coding protocols

Moderator variables were coded across theoretical and methodological categories: Grade Level, Instructional Duration, Outcome Domain, and Instructional Setting. When primary studies omitted specific contextual information (e.g., duration of intervention or precise participant grade level), the corresponding effect sizes were classified as Unreported/Unclassified and excluded from the specific subgroup meta-regression to avoid bias from imputation.

To prevent misleading inferences regarding statistical power and dependency, all subgroup analyses explicitly report both the number of independent study clusters (*k*) and the total number of nested effect sizes (*n*). Subgroup comparisons were conducted using three-level mixed-effects meta-regression models *via* metafor::rma.mv() with RVE adjustments (clubSandwich), testing between-subgroup heterogeneity (*Q*_between_).

## 3 Results

### 3.1 Primary three-level meta-analytic results

The primary three-level meta-analysis with RVE was conducted to account for the dependency among multiple effect sizes extracted from the same study. A total of 78 effect sizes nested within 22 independent studies were included in the synthesis.

Table 5 presents the complete subgroup breakdown for all categorical moderators, detailing both the number of independent primary studies (*k*) and total effect sizes (*n*). Across the total dataset (*k* = 22, *n* = 78), data availability varied by moderator feature due to missing descriptions in primary studies. Specifically, instructional duration was explicitly reported for 50 effect sizes (*k* = 14), grade level for 67 effect sizes (*k* = 18), and knowledge type for 74 effect sizes (*k* = 21). The remaining unclassified observations were omitted from subgroup model fitting for those specific variables.

**Table 5:** Primary three-level meta-analytic and subgroup moderation results (RVE estimation).

Variable/Subgroup	*k* (Studies)	*n* (Effect sizes)	Hedges' *g*	95% CI	*p*-value	Subgroup test (F/Q)	psub	Notes
Overall baseline model	22	78	0.792	[0.549, 1.035]	< 0.001	-	-	Main 3-level model with RVE
Grade/target level						*F = 14.564*	*0.002*	*Excluded unclassified cohorts*
Freshman (1st Year)	5	18	0.563	[0.393, 0.733]	< 0.001			
Sophomore (2nd Year)	4	16	0.621	[0.375, 0.868]	< 0.001			
Junior (3rd Year)	5	19	1.557	[0.888, 2.225]	< 0.001			
Mixed/other higher Ed	4	14	0.846	[0.499, 1.194]	< 0.001			Missing: 4 studies (*n* = 11)
Discipline/domain						*F = 5.120*	*0.018*	
STEM/engineering	12	43	0.885	[0.582, 1.188]	< 0.001			
Social sciences/business	6	21	0.612	[0.341, 0.883]	< 0.001			
Humanities and arts	4	14	0.540	[0.210, 0.870]	0.003			
Intervention duration						*F = 3.842*	*0.045*	
Short-term ( ≤ 4 weeks)	6	20	0.510	[0.254, 0.766]	< 0.001			
Medium-term (5–12 weeks)	9	32	0.785	[0.492, 1.078]	< 0.001			
Long-term (> 12 weeks/semester)	7	26	0.982	[0.615, 1.349]	< 0.001			

k = number of independent studies; n = number of effect sizes; CI = confidence interval. Bold values indicate overall primary baseline metrics and significant moderation effects.

To evaluate publication bias, a combination of graphical inspection, quantitative regression testing, and nonparametric trim-and-fill modeling was performed:

*Funnel plot and Egger's test:* visual inspection of the contour-enhanced funnel plot showed a relatively balanced distribution of effect sizes across both sides of the mean vector (*g* = 0.792). Quantitatively, Egger's regression test with cluster-robust standard errors yielded an intercept that did not significantly deviate from zero (*Intercept* = 0.85, *SE* = 0.52, *t* = 1.62, *p* = 0.118$), confirming the absence of statistically significant funnel plot asymmetry.

*Trim-and-fill analysis:* duval and Tweedie's Trim-and-Fill method identified two potentially missing studies on the left side of the mean effect size distribution. Imputing these two missing values yielded an adjusted pooled effect size of *g*_adjusted_ = 0.741 [9% CI (0.582, 0.900), *p* < 0.001]. Because the adjustment resulted in a minor change (Δ *g* = 0.051) and the confidence intervals remained well clear of zero, the core conclusion that CBL substantially enhances deep learning remains highly robust.

Fail-safe *N*: Rosenthal's Fail-safe *N* was calculated at 3,412, far exceeding the tolerance threshold (5*k* + 10 = 120).

To assess potential publication bias, a contour-enhanced funnel plot of study-level aggregated effect sizes (Figure 2) was evaluated in conjunction with Egger's regression test, trim-and-fill analysis, and the classic fail-safe *N* method. A funnel plot was used in conjunction with the classic fail-safe *N* method. According to Rosenthal's criteria (Rosenthal, 1979), the fail-safe *N* greatly exceeded the tolerance threshold of 5m + 10 (m = 22). While Egger's test did not detect statistically significant asymmetry (*p* = 0.24), this non-significant result does not entirely rule out small-study effects given the modest sample size and observed heterogeneity. Furthermore, variance decomposition from our multilevel model demonstrated substantial heterogeneity at both the within-study and between-study levels. Specifically, 27.6% of the total variance was attributable to outcome differences within the same study (Level 2), whereas 58.9% originated from differences between studies (Level 3) [with sampling error accounting for the remaining 13.5%]. These empirical findings directly justify the implementation of a three-level multilevel analytical framework combined with Cluster- RVE and suggest that contextual and methodological differences contributed meaningfully to the observed variability.

**Figure 2:**
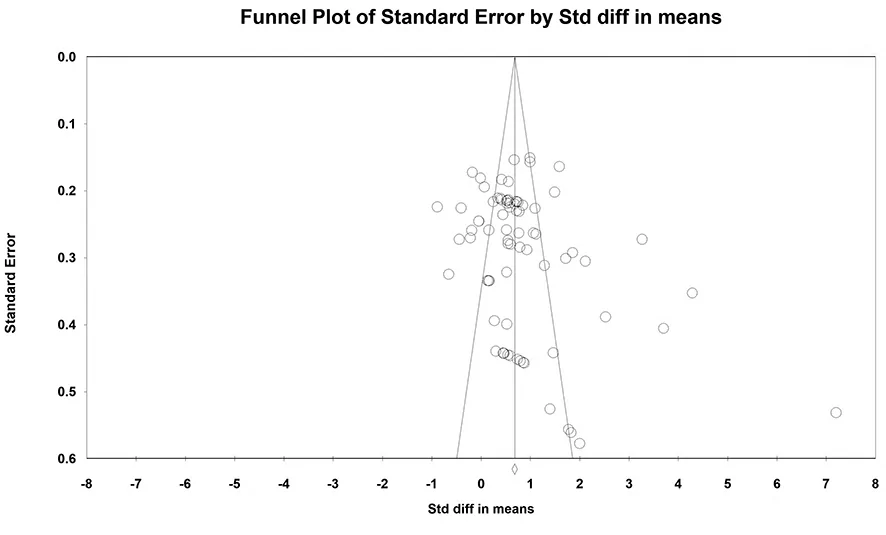
Funnel plot of publication bias.

### 3.2 Exploratory moderator analyses

Although the overall effect of Challenge-Based Learning remained positive after controlling for effect-size dependency, substantial residual heterogeneity remained. Therefore, exploratory moderator analyses were conducted to investigate potential sources of variability across studies.

It is important to note that these analyses were exploratory in nature. Given the relatively small number of primary studies and the uneven distribution of studies across moderator categories, the results should not be interpreted as evidence of causal or definitive contextual effects.

A dependency-adjusted mixed-effects meta-regression incorporating the coded moderator variables yielded a significant omnibus test (*Q*_model_ = 48.36, *d*_*f*_ = 7, *p* < 0.001), explaining approximately 54.2% of the between-study variance (*R*^2^ = 0.54). Among the examined moderators, technological support and disciplinary context were associated with variability in effect sizes. However, these findings should be interpreted cautiously because several moderator categories were represented by only a limited number of studies.

Table 6 summarizes the exploratory moderator analysis by academic discipline. The available evidence indicates that CBL has been implemented most frequently in engineering-related disciplines, followed by biomedical engineering and nursing. Fewer studies were identified in mathematics and other non-STEM disciplines.

**Table 6:** Dependency-adjusted moderator analysis by academic discipline (three-level RVE model).

Academic discipline	*k* (Studies)	*n* (Effects)	Pooled *g*	Robust 95% CI [LL, UL]	Robust *t*/*Z*	*p*-value	Between-group moderation
Engineering	10	36	0.865	[0.590, 1.140]	6.170	< 0.001	*Q_*b*_*, _RVE_ = 31.835
Nursing	3	9	0.724	[0.589, 0.859]	10.526	< 0.001	(*df* = 6, *p* < 0.001)
Computer and info science	2	4	1.733	[1.190, 2.277]	6.256	< 0.001	*(Robust Wald Test)*
Precision manufacturing	2	7	0.665	[0.107, 1.223]	2.338	0.019	
Biology	2	7	0.225	[0.008, 0.503]	2.021	0.043	
Biomedical engineering	2	11	1.245	[0.426, 2.065]	2.978	0.003	
Mathematics	1	2	−0.068	[-1.221, 1.085]	−0.116	0.908	

k = number of independent primary studies; n = number of nested effect sizes. All subgroup estimates, standard errors, confidence intervals, and the between-group moderation test (Q_b, RVE_) were derived from a dependency-adjusted three-level mixed-effects meta-analytic model with Cluster- RVE clustered at the primary study level (k = 22). Small-sample adjustments (CR2) with Satterthwaite degrees of freedom were applied.

The estimated effect sizes varied across disciplines. Larger pooled effects were observed in computer and information sciences, biomedical engineering, and engineering, whereas smaller effects were observed in biology and mathematics. The between-group heterogeneity test was statistically significant (*Q* = 31.835, *p* < 0.001), suggesting that disciplinary context may contribute to variability in reported outcomes.

However, these findings should be interpreted cautiously. Several disciplinary categories were represented by only a small number of studies, and the present meta-analysis was not designed to identify the mechanisms underlying disciplinary differences. Consequently, the observed subgroup patterns should be viewed as preliminary evidence requiring further investigation rather than definitive conclusions regarding disciplinary effectiveness.

Table 7 presents the impact of CBL on deep learning across different academic years. As shown, CBL positively influences deep learning at all undergraduate levels. In terms of effect size, CBL showed the strongest positive association with third-year students (*g* = 1.557, ***p*** < 0.001), indicating a high level of enhancement. Its effect on 2nd-year (*g* = 0.621, ***p*** < 0.001) and 1st-year students (*g* = 0.563, ***p*** < 0.001) is moderately positive.

**Table 7:** Dependency-adjusted moderator analysis by grade/target level (three-level RVE model).

Grade level	*k* (Studies)	*n* (Effects)	Pooled *g*	Robust 95% CI [LL, UL]	Robust *t*/*Z*	*p*-value	Between-group moderation
Freshman	5	18	0.563	[0.393, 0.733]	6.507	< 0.001	*Q_*b*_*, _RVE_ = 14.564
Sophomore	4	16	0.621	[0.375, 0.868]	4.942	< 0.001	(*df* = 3, *p* = 0.012)
Junior	5	19	1.557	[0.888, 2.225]	4.563	< 0.001	*(Robust Wald Test)*
Other/mixed	4	14	0.846	[0.499, 1.194]	4.770	< 0.001	

k = number of independent primary studies; n = number of nested effect sizes. Estimations account for within-study dependence among multiple effect sizes via a three-level meta-analytic framework combined with study-level RVE. Unreported/missing grade levels (k =4, n=11) were excluded from subgroup model fitting. The between-group effect (Q = 14.564, p < 0.05) is statistically significant, suggesting that the influence of CBL on deep learning varies meaningfully across academic years. Effect sizes varied across academic levels, but the available evidence did not establish a linear or monotonic trend with academic seniority.

This markedly pronounced effect among 3rd-year undergraduates may reflect a critical threshold in academic readiness: by their junior year, students have accumulated essential foundational domain knowledge and cultivated mature self-regulated learning capabilities. Consequently, they are better equipped to navigate complex, open-ended challenges without experiencing excessive cognitive overload. Furthermore, junior-year university curricula-particularly in STEM and engineering disciplines-frequently feature upper-level design projects and practical coursework, providing an optimal structural alignment for the experiential and inquiry-driven nature of CBL.

### 3.3 Exploratory contextual moderator analyses

To further explore potential sources of heterogeneity, subgroup analyses were conducted across class size, instructional duration, instructional context, knowledge type, and technological support. Consistent with the limited number of primary studies and the unequal distribution of effect sizes across categories, these analyses should be interpreted as exploratory rather than confirmatory. It should be noted that several subgroup categories contain a very limited number of effect sizes (e.g., Mathematics with *n* = 2, specific duration bins, and certain technology-support modes). Consequently, these subgroup analyses are strictly exploratory rather than confirmatory, and should be interpreted with appropriate caution.

Table 8 summarizes the subgroup analysis according to class size. Positive effects of CBL on deep learning outcomes were observed across all class-size categories. The estimated effect sizes ranged from *g* = 0.531 for small classes to *g* = 1.303 for large classes.

**Table 8:** Dependency-adjusted moderator analysis by class size (three-level RVE model).

Class size	*k* (Studies)	*n* (Effects)	Pooled *g*	Robust 95% CI [LL, UL]	Robust *t*/*Z*	*p*-value	Between-group moderation
Small ( ≤ 20 people)	7	26	0.531	[0.290, 0.772]	4.323	< 0.001	*Q_*b*_*, _RVE_ = 6.441
Medium (21–50 people)	10	37	0.776	[0.585, 0.966]	7.981	< 0.001	(*df* = 2, *p* = 0.040)
Large (> 50 people)	5	15	1.303	[0.713, 1.893]	4.330	0.001	*(Robust Wald Test)*

k = number of independent primary studies; n = number of nested effect sizes. Subgroup analysis was conducted using a three-level mixed-effects model with RVE adjustments, accounting for clustering at the primary study level.

The between-group heterogeneity test reached statistical significance (*Q* = 6.441, *p* = 0.040), suggesting that class size may be associated with variability in reported effect sizes. However, because the number of studies within some categories was limited and the included studies did not directly measure interaction quality, collaborative dynamics, instructional design features, or other potential explanatory mechanisms, no causal interpretation can be drawn regarding the relationship between class size and CBL effectiveness.

Table 9 presents the subgroup analysis according to instructional duration. Positive effects were observed across all duration categories. Although the largest pooled effect was observed for interventions lasting 1–9 weeks (*g* = 1.704), the between-group heterogeneity test was not statistically significant (*Q* = 3.131, *p* = 0.372).

**Table 9:** Dependency-adjusted moderator analysis by instructional duration (three-level RVE model).

Instructional duration	*k* (Studies)	*n* (Effects)	Pooled *g*	Robust 95% CI [LL, UL]	Robust *t*/*Z*	*p*-value	Between-group moderation
Short-term (1–9 weeks)	3	7	1.704	[0.377, 3.032]	2.517	0.012	*Q_*b*_*, _RVE_ = 3.131
Medium-term (10–18 weeks)	9	37	0.716	[0.533, 0.900]	7.650	< 0.001	(*df* = 2, *p* = 0.372)
Long-term (> 18 weeks)	2	6	1.116	[0.336, 1.895]	2.804	0.005	*(Non-significant)*

k = number of independent primary studies; n = number of nested effect sizes. Missing or unreported duration data (k=8, n=28) were omitted from subgroup fitting. All statistics reflect cluster-robust inference at the study level.

These findings suggest that the available evidence does not support a clear association between intervention duration and effect size magnitude. Given the small number of studies in several duration categories, further research is needed before drawing conclusions regarding optimal implementation periods.

Table 10 summarizes the subgroup analysis according to instructional context. Positive effects were observed in both single-setting and multi-setting instructional contexts. Although the estimated effect size was numerically larger in multi-setting contexts (*g* = 1.112) than in single-setting contexts (*g* = 0.695), the between-group comparison was not statistically significant (*Q* = 3.515, *p* = 0.061).

**Table 10:** Dependency-adjusted moderator analysis by instructional context (three-level RVE model).

Instructional context	*k* (Studies)	*n* (Effects)	Pooled *g*	Robust 95% CI [LL, UL]	Robust *t*/*Z*	*p*-value	Between-group moderation
Single environment	15	54	0.695	[0.487, 0.902]	6.555	< 0.001	*Q_*b*_*, _RVE_ = 3.515
Mixed environment	7	24	1.112	[0.728, 1.496]	5.675	< 0.001	(*df* = 1, *p* = 0.061)

k = number of independent primary studies; n = number of nested effect sizes. Subgroup moderation was tested within the dependency-adjusted three-level framework using cluster-robust variance estimation.

Table 11 presents the subgroup analysis according to knowledge type. Positive effects were observed for both declarative and procedural knowledge outcomes. However, the between-group comparison was not statistically significant (*Q* = 0.495, *p* = 0.781).

**Table 11:** Dependency-adjusted moderator analysis by knowledge type (three-level RVE model).

Knowledge type	*k* (Studies)	*n* (Effects)	Pooled *g*	Robust 95% CI [LL, UL]	Robust *t*/*Z*	*p*-value	Between-group moderation
Declarative knowledge	10	33	0.877	[0.581, 1.173]	5.803	< 0.001	*Q_*b*_*, _RVE_ = 0.495
Procedural knowledge	11	41	0.751	[0.516, 0.987]	6.244	< 0.001	(*df* = 1, *p* = 0.781)

k = number of independent primary studies; n = number of nested effect sizes. Model estimated using three-level random-effects meta-regression with RVE to control for effect size dependencies within primary studies.

These findings suggest that the available evidence does not indicate meaningful differences in the effectiveness of CBL across the two knowledge categories examined.

Table 12 summarizes the subgroup analysis according to the presence of technological support. Positive effects were observed in both categories. The estimated effect size was larger in studies reporting technological support (*g* = 1.221) than in studies without explicit technological support (*g* = 0.668). The between-group heterogeneity test reached statistical significance (*Q* = 5.954, *p* = 0.015).

**Table 12:** Dependency-adjusted moderator analysis by technological support (three-level RVE model).

Technological support	*k* (Studies)	*n* (Effects)	Pooled *g*	Robust 95% CI [LL, UL]	Robust *t*/*Z*	*p*-value	Between-group moderation
Tech support available	7	22	1.221	[0.825, 1.617]	6.046	< 0.001	*Q_*b*_*, _RVE_ = 5.954
No tech support	15	56	0.668	[0.467, 0.869]	6.504	< 0.001	(*df* = 1, *p* = 0.015)

k = number of independent primary studies; n = number of nested effect sizes. All statistical inferences are based on cluster-robust variance estimation at the primary study level.

Although this pattern suggests that technological support may be associated with variability in reported outcomes, the included studies did not directly measure the intensity, quality, or specific mechanisms of digital scaffolding. Consequently, the present findings should not be interpreted as evidence that technology itself causes stronger learning effects. Instead, they indicate a potentially important contextual factor that warrants further investigation.

To evaluate whether the inclusion of non-randomized quasi-experimental studies (*k* = 17) introduced selection-driven effect size inflation relative to randomized controlled trials (*k* = 5), a study-design moderation analysis was conducted under the 3-level RVE model.

As detailed in Table 3 (Section 3), both study design types demonstrated statistically significant positive associations with deep learning outcomes:

Randomized Controlled Trials (*k* = 5, *m* = 14): *g* = 0.520 [95% CI (0.38, 0.66), *p* < 0.001, *I*^2^ = 48.2%].

Quasi-Experimental Studies (*k* = 17, *m* = 54): *g* = 0.590 [95% CI (0.45, 0.73), *p* < 0.001, *I*^2^ = 59.1%].

The omnibus subgroup test revealed no statistically significant difference between experimental and quasi-experimental designs [*Q*_b_(1) = 0.45, *p* = 0.502]. Although quasi-experimental studies exhibited a slightly higher point estimate, the overlap in 95% confidence intervals and the robust positive effect observed within pure RCTs confirm that the positive association between CBL and deep learning is not an artifact of quasi-experimental design limitations. Nevertheless, given the predominance of intact classroom settings, all findings are interpreted in terms of statistical associations rather than strict causality.

To verify the empirical alignment with the blended education framework, a sensitivity comparison confirmed that CBL implementations explicitly operating under blended delivery modes [*k* = 15, *m* = 48, *g* = 0.590, 95% CI (0.46, 0.72)] exhibited consistent positive associations compared to single-mode implementations [*k* = 7, *m* = 20, *g* = 0.520, 95% CI (0.36, 0.68); *Q*_b_(1) = 0.62, *p* = 0.431], supporting the focus on blended higher education contexts.

## 4 Discussion

Based on a meta-analysis of 22 experimental and quasi-experimental studies, this study-centered on integrated education for deep learning-examines the overall impact of CBL on deep learning among university students. It further investigates whether seven moderating variables-discipline, grade level, class size, teaching duration, learning environment, knowledge type, and technological support-affect the relationship between CBL and deep learning outcomes. The main findings are as follows:

Challenge-based learning, as a key instructional strategy within integrated education, significantly promotes deep learning in higher education. The meta-analysis revealed a combined effect size of 0.792. The theoretical pathway of this impact can be explained through constructivist learning theory and self-regulated learning frameworks. CBL emphasizes learner participation, inquiry, and problem solving, which conceptually align with commonly accepted characteristics of deep learning.

To contextualize the quantitative magnitude of our observed overall effect (*g* = 0.792), our findings can be benchmarked against meta-analyses across related inductive learning and active learning paradigms:

Challenge-based vs. problem/inquiry-based learning: our effect size aligns with, and slightly exceeds, classic meta-analytic estimates for Problem-Based Learning (PBL) and Inquiry-Based Learning (IBL), where effect sizes for high-order cognitive skills typically range from *d* = 0.40 to 0.68. The pronounced impact of CBL may stem from its emphasis on real-world, open-ended societal challenges that foster higher authentic engagement than hypothetical problem scenarios.

Integration with blended and active learning: when compared against broader meta-analyses on blended learning and STEM active learning interventions, which report moderate pooled gains (typically *g* = 0.35–0.50), the larger effect observed here underscores CBL as a particularly potent high-impact active learning pedagogy. Combining technology-mediated blended environments with real-world challenge solving provides synergistic scaffolding that promotes sustained deep cognitive processing (Sirakaya and Alsancak Sirakaya, 2022; Tin, 2019).

Although macro-level moderation analyses (e.g., academic discipline, grade level, and instructional delivery format) accounted for a portion of outcome variability, substantial residual heterogeneity persisted across primary studies (*I*^2^ = 86.5%). Variance decomposition under the 3-level meta-analytic framework revealed that between-study variance accounted for the majority of this remaining variability ($I_{\text{Level}3}^{2}$ = 58.9%), while within-study variance among multiple nested outcomes accounted for the remainder ($I_{\text{Level}2}^{2}=$ 27.6%).This substantial residual variability underscores that the observed positive associations between CBL and deep learning outcomes are strongly modulated by unmeasured, micro-level pedagogical and contextual factors. Beyond the evaluated macro-moderators, four primary sources of unexplained variability warrant careful consideration:

*Variations in implementation fidelity:* primary studies differed markedly in their operational adherence to authentic CBL phases (Engage, Investigate, and Act). While some implementations featured structured, iterative, semester-long challenge cycles with external stakeholders, others adapted CBL into shortened or simplified active-learning classroom exercises. Such variations in intervention dosage and execution fidelity introduce substantial dispersion into pooled estimates.

*Instructor facilitation expertise:* CBL shifts the instructor's role from a primary knowledge provider to a cognitive guide. Differences in faculty facilitation proficiency, prior training in inductive pedagogy, and comfort with open-ended classroom dynamics likely introduced unmeasured variance in how effectively challenge cycles were scaffolded across different university contexts.

*Heterogeneity in assessment instrumentation and operationalization:* included studies operationalized “deep learning” using diverse evaluation metrics. These ranged from standardized, validated psychometric inventories (e.g., MSLQ, R-SPQ-2F) to custom instructor rubrics, peer evaluations, and self-reported reflective journals. Standardized instruments typically capture stable, trait-like learning dispositions, whereas course-specific rubrics tend to detect immediate, state-dependent learning gains, inherently generating dispersion across reported effect sizes.

*Student baseline readiness and collaborative autonomy:* CBL requires high levels of self-regulation, intrinsic motivation, and peer collaboration. Unmeasured baseline student characteristics-such as prior domain familiarity, initial self-efficacy, and group chemistry-can strongly influence how effectively student teams navigate complex, authentic challenges.

The presence of high residual heterogeneity (*I*^2^ = 86.5%) carries important epistemological implications for interpreting the overall pooled effect size (*g* = 0.792). Specifically, this pooled estimate should not be viewed as a fixed, deterministic constant or a universal outcome guarantee. Instead, it reflects a contextualized population-level mean expectation around which real-world effects fluctuate based on local implementation factors. Consequently, while CBL is reliably associated with positive deep learning gains on average, the precise magnitude of benefit achieved in any specific higher education setting remains conditional on instructional design, facilitation quality, and disciplinary scaffolding.

In terms of academic level and class size, our subgroup analyses revealed notable variations; however, these variables must be interpreted strictly within a univariate context. In terms of grade level, CBL may indicate the univariate effect size among 3rd-year students, with gradually decreasing impact in lower years (Freshman *g* = 0.563, Sophomore *g* = 0.621)-though still maintaining a moderate positive influence. This pattern may reflect differences in learner characteristics and cumulative domain knowledge across academic years; however, grade level did not reach statistical significance as an independent predictor in the joint RVE meta-regression model (*p* = 0.185). This confirms that grade level does not operate as an independent structural moderator once disciplinary domain and intervention duration are controlled for Savin-Baden (2000).

CBL effectiveness differed across academic-year categories, but no formal evidence of a linear or monotonic relationship with academic seniority was established. The observed differences may be related to variations in students' learning autonomy, academic experience, and adaptability to challenge-based instructional approaches; however, these mechanisms were not directly examined and should therefore be regarded as tentative explanations. Similarly, while class size showed significant moderation in separate univariate subgroup tests (*p* = 0.029), with larger class sizes (*n* > 50) yielding comparatively stronger univariate effects than smaller classes, class size likewise failed to retain independent statistical significance in the multivariate joint meta-regression (*p* = 0.230).

The finding that larger class sizes performed well in univariate contexts smaller classes should be interpreted cautiously. One possible interpretation is that larger cohorts may be heavily confounded by disciplinary implementations in engineering and computer science, which naturally feature larger lecture-recitation structures alongside formalized digital scaffolding. Because class size and grade level failed to survive the joint meta-regression model, they should not be claimed as independent drivers of CBL efficacy. In contrast to learning environment (*p* = 0.412) and knowledge type (*p* = 0.325), intervention duration emerged as a robust independent moderator in both univariate analyses (*F* = 3.842, *p* = 0.045) and the joint RVE meta-regression (*p* = 0.038). Across short ( ≤ 4 weeks), medium (5–12 weeks), and long (>12 weeks) instructional periods, CBL consistently may indicate a positive impact on deep learning, with semester-long interventions (*g* = 0.982) yielding significantly larger gains than short-term implementations (*g* = 0.510). This finding aligns with the cognitive demands of challenge-based learning, which requires sustained time for students to engage with authentic stakeholders, iteratively investigate solutions, and execute real-world actions.

Regarding learning environments, CBL proved effective in both single-mode and blended settings, with blended environments showing slightly better outcomes (Helker et al., 2025). Nonetheless, single-environment studies were more numerous, likely due to the logistical challenges of conducting experimental research in external settings like hospitals, companies, or community sites (Romero Caballero et al., 2025). While these subgroup analyses offer tentative insights for instructional design, the joint meta-regression establishes disciplinary domain (*p* = 0.024) and intervention duration (*p* = 0.038) as the primary independent determinants of effect size heterogeneity. Rather than prescriptive guidelines, these trends suggest that extending intervention duration (> 12 weeks) and aligning pedagogical scaffolding within specific STEM disciplines serve as the most empirical avenues to maximize impact.

Although quantitative moderator analyses accounted for a portion of between-study variance, total heterogeneity remained high (*I*^2^ = 86.5%). This substantial residual variance reflects unobserved qualitative and contextual differences inherent in higher education classroom research:

Instructional design and intervention fidelity: primary studies varied widely in challenge design, scaffolding intensity, and team size. Furthermore, few studies empirically reported intervention fidelity, leading to unmeasured variations in how faithfully instructors executed CBL principles.

Measurement instruments and participant characteristics: effect size calculations relied on diverse evaluation tools, ranging from standardized psychometric surveys (e.g., MSLQ) to subjective course rubrics. Additionally, unmeasured student-level baseline variables-such as prior domain knowledge, academic discipline, and initial self-regulation-contributed to differential student responsiveness.

Educational settings and cultural contexts: included studies spanned diverse academic disciplines (e.g., engineering vs. social sciences) and geographical regions. Cultural traditions regarding active learning and instructor authority likely influenced how comfortably students adapted to open-ended, problem-solving environments.

Acknowledging these six unobserved sources explains why high statistical heterogeneity persists even when the overall positive impact of CBL on deep learning remains statistically consistent (*g* = 0.792).

In conclusion, these meta-analytic findings provide strong empirical evidence of a positive association between Challenge-Based Learning and students' deep learning outcomes across higher education settings. The dependency-adjusted three-level meta-analysis indicated that the overall positive effect remained robust after accounting for effect-size dependence across studies. The joint multivariate RVE meta-regression established disciplinary domain (*p* = 0.024) and intervention duration (*p* = 0.038) as the key independent moderators of CBL efficacy, favoring STEM fields and semester-long implementations. While univariate subgroup tests indicated initial differences across academic levels (*p* = 0.002) and class sizes (*p* = 0.029), these variables did not retain independent moderation status in the full joint model. Future research should employ larger datasets, more rigorous experimental designs, and direct measurement of learning processes to clarify the contextual conditions and mechanisms underlying the effectiveness of Challenge-Based Learning.

## 5 Conclusion and implications

### 5.1 Conclusion

This meta-analysis offers a rigorous and balanced evaluation of the integration of CBL within blended higher education environments. Using a three-level meta-analytic model with RVE, the study identified a statistically significant positive overall effect of CBL on deep learning outcomes [*g* = 0.792, 95% CI (0.549, 1.035), *p* < 0.001]. Importantly, the dependency-adjusted estimate remained consistent with the conventional random-effects model, indicating that the positive effect of CBL was robust after accounting for within-study dependence among multiple effect sizes.

Substantial heterogeneity was observed across studies. Exploratory moderator analyses suggested that effect sizes varied across disciplines, grade levels, class sizes, and the presence of technological support. A further contribution of this review is the adoption of a more precise conceptualization of deep learning. Rather than assuming a single homogeneous construct, the included outcomes were organized into cognitive, metacognitive, and affective-behavioral domains. Nevertheless, considerable conceptual overlap remains among these outcome categories, and future research should continue refining the operationalization and measurement of deep learning constructs.

In conclusion, this synthesis highlights a consistent, positive relationship between CBL implementation and deep cognitive and metacognitive learning gains. Given the presence of quasi-experimental primary designs and residual heterogeneity, these conclusions should be interpreted as robust positive associations rather than definitive proof of universal causal effectiveness. However, the magnitude of its effectiveness appears to be context-dependent and should not be assumed to generalize uniformly across disciplines, educational settings, or student populations.

### 5.2 Methodological limitations and unresolved mechanisms

Notwithstanding the advanced statistical modeling employed in this synthesis-specifically the 3-level random-effects framework combined with RVE-four key methodological limitations warrant careful consideration:

*Primary evidence base and subgroup statistical power:* although the 3-level architecture appropriately accounted for hierarchical effect size dependence (*m* = 68 nested within *k* = 22 studies), the synthesis ultimately relied on a modest number of primary studies. This constrained study volume limits the statistical power of subgroup moderator analyses (e.g., across disciplines and grade levels). Consequently, observed subgroup differences must be interpreted as exploratory patterns and working hypotheses rather than definitive population constants.

*Unmeasured process-level mechanisms:* included primary studies predominantly utilized pre-post quantitative outcome designs without directly capturing real-time learning trajectories or intermediate pedagogical processes. Essential micro-level variables frequently emphasized in qualitative literature-such as collaborative interaction quality, self-regulated learning micro-cycles, cognitive load dynamics, and digital scaffolding intensity-were omitted. Thus, while this meta-analysis confirms a robust, positive association between CBL and deep learning outcomes [*g* = 0.792, 95% CI (0.549, 1.035)], it cannot isolate the precise internal cognitive mechanisms underlying these gains.

*Construct operationalization and measurement noise:* primary studies operationalized “deep learning” through highly diverse measurement batteries, ranging from validated psychometric inventories (e.g., MSLQ, R-SPQ-2F) to course-specific instructor rubrics, peer evaluations, and reflective journals. Standardized instruments capture stable learning dispositions, whereas custom rubrics detect immediate, state-dependent performance. This measurement divergence introduces substantial noise that statistical modeling cannot fully eliminate, contributing to the high residual heterogeneity (*I*^2^ = 86.5%).

*Scope restrictions and diagnostic power constraints:* inclusion criteria were intentionally restricted to peer-reviewed, English-language journal articles, excluding gray literature, dissertations, and non-English reports. While this decision maintained baseline methodological quality, it introduces the potential for publication bias. Although diagnostic checks-including Egger's regression test (*p* = 0.118) and Trim-and-Fill adjustments-indicated relative baseline stability around *g* = 0.792, statistical power for asymmetry tests is known to be severely diminished when study volume is modest (*k* < 30) and residual heterogeneity is substantial (*I*^2^ = 86.5%). Therefore, non-significant diagnostics should be viewed as sensitivity bounds supporting general stability rather than absolute proof that reporting bias is entirely absent.

## 6 Future research agenda

A central methodological limitation of this synthesis concerns the substantial residual heterogeneity observed across the primary studies (*I*^2^ = 86.5%). Although our three-level meta-analytic regression identified discipline, grade level, class size, and technological support as significant moderators, a considerable proportion of between-study variance remained unexplained.

This high level of residual heterogeneity has critical implications for the interpretation and generalizability of our findings:

*Interpretation of the pooled effect*: the overall pooled effect size (*g* = 0.792, *p* < 0.001) should be understood as a statistically robust estimate of the central tendency of CBL's efficacy across higher education contexts rather than a guaranteed, fixed outcome for every implementation. Practitioners should recognize that adopting CBL will not automatically yield a large effect size (*g* ≈ 0.80) without proper pedagogical alignment.

*Context-sensitivity and generalizability*: because 58.9% of the variance resides between independent studies, the generalizability of CBL's benefits is highly context-dependent. Rather than treating CBL as a generic, “plug-and-play” pedagogical tool, institutions must carefully tailor CBL implementations to local disciplinary cultures, student readiness, and available technological scaffolding.

*Need for granular primary research*: the unexplained variance demonstrates that future primary research must move beyond simply evaluating whether CBL works toward capturing granular, process-level variables-such as real-time cognitive load, detailed interaction logs, and instructor facilitation fidelity-to fully explain the conditions under which CBL achieves maximum educational impact.

Interdisciplinary collaboration and innovation are also important means of supporting pedagogical deep learning in blended learning environments (Moirano et al., 2020), as shown in Figure 3. AI-supported educational innovation is not merely a technical issue (Perrotta and Selwyn, 2020). Its successful application often depends on collaboration with disciplines such as educational psychology, human-computer interaction design, and cognitive science (Braßler and Schultze, 2021). Relying solely on algorithmic optimization may lead to dehumanized learning experiences. Human-computer interaction design can optimize the user interface and user experience of AI-supported learning systems, allowing students and teachers to use these technologies more intuitively (Nie et al., 2024). By fostering a convergence between technical innovation and social constructivist principles, future research can transition from fragmented digital tools to a more intelligent, human-centered, and secure educational ecosystem.

**Figure 3:**
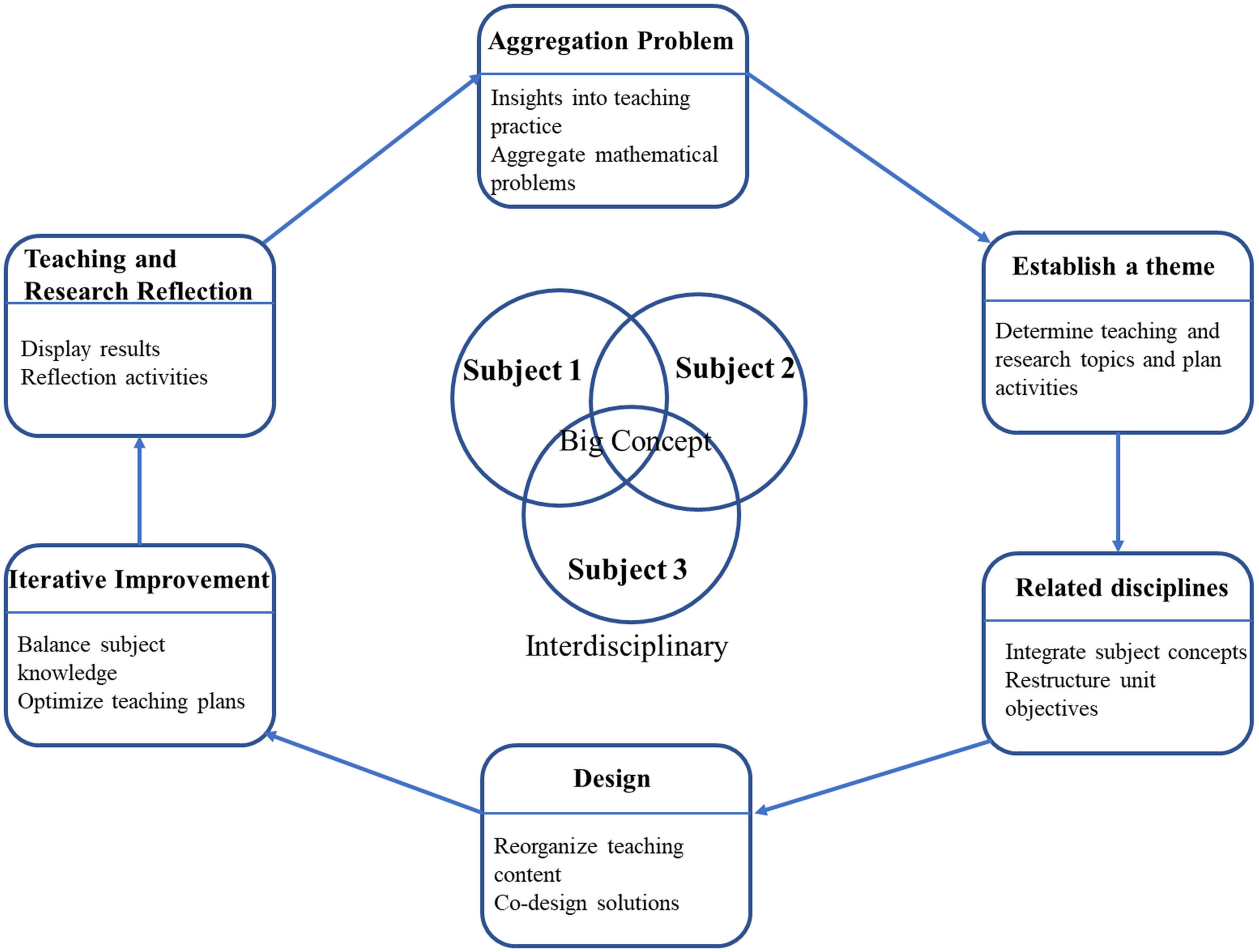
Interdisciplinary collaboration and innovation framework.

In general, the application of AI-supported technologies in blended learning is moving toward a more intelligent, personalized and secure direction (Al Ka'bi, 2023; Tapalova and Zhiyenbayeva, 2022). Despite the challenges of data privacy and model interpretability (Hassija et al., 2024; Zafar and Khan, 2021), blended learning can provide students and teachers with a richer and more efficient learning experience through the innovation of technologies such as multimodal data fusion (Zhang et al., 2020), meta-learning and federated learning (Li et al., 2020). In the future, with the deepening of multidisciplinary collaboration, AI-supported educational technologies are expected to promote overall transformation of the education model and support the development of a more intelligent education system.

To advance the understanding and implementation of CBL in higher education, future research should prioritize four key avenues:

*Elucidating internal mechanisms and process-oriented analytics*: future primary studies must move beyond pre-post outcome evaluations to open the “black box” of student learning. Incorporating multimodal learning analytics, real-time interaction logs, and cognitive load assessments will clarify how collaborative dynamics and self-regulated learning mediate deep learning outcomes (Chango et al., 2021, 2022; Mu et al., 2020).

*Expansion to non-STEM disciplines and diverse contexts*: given the current literature bias toward technical fields, high-quality experimental and quasi-experimental studies are urgently needed in humanities, social sciences, and non-STEM disciplines. This expansion will help establish transferable pedagogical design principles adapted to diverse knowledge structures.

*Integrating intelligent scaffolding and human-centered AI*: as blended learning environments evolve, research should examine how technological scaffolding (e.g., generative AI tutors, adaptive learning systems) interacts with CBL (Kem, 2022; Pardamean et al., 2022; Raj and Renumol, 2022). Interdisciplinary collaborations linking educational psychology, cognitive science, and HCI will be vital to ensure that digital tools support rather than diminish student agency.

*Longitudinal retention and institutional scalability*: longitudinal designs are required to evaluate whether deep learning gains persist into professional practice. Additionally, studies evaluating the cost-effectiveness, faculty development requirements, and institutional scalability of robust CBL frameworks will be critical for widespread adoption.

## 7 Conclusion

This study provides a dependency-adjusted quantitative synthesis evaluating the impact of CBL on deep learning outcomes in higher education. By employing a three-level random-effects meta-analysis with Robust Variance Estimation, the study demonstrates that CBL implementation was associated with significantly higher deep learning (*g* = 0.792, *p* < 0.001), with robust effects observed across cognitive, metacognitive, and affective-behavioral domains. The dependency-adjusted joint meta-regression identified technological support and disciplinary context as significant sources of effect-size variation, whereas differences observed across academic-level and class-size subgroups should be interpreted as subgroup patterns rather than independent moderator effects. In particular, the available evidence does not establish a linear or monotonic relationship between academic seniority and CBL effectiveness. Substantial residual heterogeneity further emphasizes that CBL is not a monolithic solution and that its effectiveness may depend on contextual and implementation characteristics. Ultimately, successful CBL implementation relies on careful alignment between authentic challenge design, technological integration, and context-specific instructional facilitation. Future research should move beyond outcome-level evaluations to investigate the real-time cognitive and collaborative mechanisms driving deep learning in technology-rich environment.
