# Supplementary material for: Challenge-based learning in blended education: a meta-analysis of deep learning outcomes
Source: Front Psychol. 2026 Sep 23;17:1939274. doi: 10.3389/fpsyg.2026.1939274 (PMC13643514; doi:10.3389/fpsyg.2026.1939274)
Supplement: Supplementary file 1 [file Supplementary_File_1.docx]

**Supplementary Appendix A**

**Table A1. Database-Specific Search Strings, Field Tags, and Applied Filters**

| Database | Exact Search String | Field Tags & Filters |
| --- | --- | --- |
| Web of Science Core Collection | TS = (("challenge-based learning" OR "challenge based learning" OR "CBL") AND ("deep learning" OR "deep approach*" OR "critical thinking" OR "metacognit*" OR "problem solving" OR "self-regulat*") AND ("higher education" OR "undergraduat*" OR "university" OR "college*")) | Topic (TS), Publication Year ≤2024, Document Types: Article, Early Access, Proceedings Paper. |
| Scopus | TITLE-ABS-KEY ( ( "challenge-based learning" OR "challenge based learning" OR "CBL" ) AND ( "deep learning" OR "deep approach*" OR "critical thinking" OR "metacognit*" OR "problem solving" ) AND ( "higher education" OR "undergraduat*" OR "university" ) ) | Title, Abstract, Keyword. Limit to English language. |
| ERIC (EBSCOhost) | ((DE "Challenge Based Learning" OR TI "challenge-based learning" OR AB "challenge-based learning") AND (DE "Deep Learning" OR AB "deep approach" OR AB "critical thinking" OR AB "metacognit*")) | Descriptors (DE), Title (TI), Abstract (AB). Educational level: Higher Education. |
| PsycINFO | ((Any Field: "challenge-based learning" OR "CBL") AND (Any Field: "deep learning" OR "critical thinking" OR "self-regulation") AND (Any Field: "higher education" OR "undergraduate")) | All fields. Population: Human; Methodology: Empirical Study. |
| IEEE Xplore | ("All Metadata": "challenge-based learning" OR "CBL") AND ("All Metadata": "deep learning" OR "education" OR "student learning") | All Metadata. Filter by Content Type: Journals, Conference Publications. |

Figure A1. PRISMA 2020 Flow Diagram for Study Selection

Table A2. Full Study-Level Leave-One-Out (LOO) Sensitivity Analysis (*k* = 22)

| Iteration | Excluded Primary Study | Remaining Studies (*k*) | Remaining Effects (*n*) | Recalculated Effect (*g*) | SE | 95% CI | *p*-value | I^2^ (%) |
| --- | --- | --- | --- | --- | --- | --- | --- | --- |
| 1 | Shakila et al. (2021) | 21 | 75 | 0.790 | 0.085 | [0.623, 0.957] | < 0.001 | 86.3 |
| 2 | Gutiérrez-Martínez et al. (2021) | 21 | 74 | 0.795 | 0.086 | [0.626, 0.964] | < 0.001 | 86.4 |
| 3 | Colombelli et al. (2022) | 21 | 75 | 0.788 | 0.085 | [0.621, 0.955] | < 0.001 | 86.2 |
| 4 | Acosta et al. (2018) | 21 | 76 | 0.793 | 0.085 | [0.626, 0.960] | < 0.001 | 86.5 |
| 5 | Fidalgo-Blanco et al. (2019) | 21 | 74 | 0.784 | 0.084 | [0.619, 0.949] | < 0.001 | 86.0 |
| 6 | Garcia-Garcia et al. (2026)* | 21 | 74 | 0.779 | 0.084 | [0.614, 0.944] | < 0.001 | 85.8 |
| 7 | Gómez et al. (2022) | 21 | 75 | 0.791 | 0.085 | [0.624, 0.958] | < 0.001 | 86.4 |
| 8 | Gómez-Puente and Doulougeri (2022) | 21 | 75 | 0.796 | 0.086 | [0.627, 0.965] | < 0.001 | 86.6 |
| 9 | Kohn Rådberg et al. (2020) | 21 | 73 | 0.768 | 0.085 | [0.601, 0.935] | < 0.001 | 85.9 |
| 10 | Leijon et al. (2022) | 21 | 75 | 0.789 | 0.085 | [0.622, 0.956] | < 0.001 | 86.3 |
| 11 | Malmqvist et al. (2015) | 21 | 74 | 0.794 | 0.086 | [0.625, 0.963] | < 0.001 | 86.4 |
| 12 | Membrillo-Hernández et al. (2017) | 21 | 72 | 0.812 | 0.086 | [0.643, 0.981] | < 0.001 | 86.2 |
| 13 | Membrillo-Hernández et al. (2021)* | 21 | 75 | 0.783 | 0.084 | [0.618, 0.948] | < 0.001 | 86.1 |
| 14 | Nowell et al. (2020) | 21 | 75 | 0.792 | 0.085 | [0.625, 0.959] | < 0.001 | 86.5 |
| 15 | Olivares et al. (2021) | 21 | 74 | 0.787 | 0.085 | [0.620, 0.954] | < 0.001 | 86.1 |
| 16 | Chapel et al. (2021) | 21 | 75 | 0.790 | 0.085 | [0.623, 0.957] | < 0.001 | 86.3 |
| 17 | Nascimento et al. (2022)* | 21 | 74 | 0.782 | 0.084 | [0.617, 0.947] | < 0.001 | 85.9 |
| 18 | Tang & Chow (2021) | 21 | 74 | 0.797 | 0.086 | [0.628, 0.966] | < 0.001 | 86.5 |
| 19 | Félix-Herrán et al. (2022) | 21 | 75 | 0.789 | 0.085 | [0.622, 0.956] | < 0.001 | 86.2 |
| 20 | Huesca et al. (2024) | 21 | 75 | 0.793 | 0.085 | [0.626, 0.960] | < 0.001 | 86.4 |
| 21 | Kong et al. (2021) | 21 | 74 | 0.786 | 0.084 | [0.621, 0.951] | < 0.001 | 86.1 |
| 22 | Zhao et al. (2021) | 21 | 75 | 0.791 | 0.085 | [0.624, 0.958] | < 0.001 | 86.4 |

*Denotes primary studies identified with an overall high risk of bias and excluded in the RoB Sensitivity Model.

**Table A3. Comprehensive Characteristics and Effect Size Extraction Table (*k* = 22, *n* = 78)**

| EffectID | StudyID | Author & Year | Discipline | Grade / Target | Study Design | Outcome Variable | Nexp / Ncon(Ntotal) | Descriptive Statistics (Mean / SD) | Test Statistics (t/F/p) | Effect Size / Finding Description | Outcome Domain |
| --- | --- | --- | --- | --- | --- | --- | --- | --- | --- | --- | --- |
| 1 | 1 | Roselli & Brophy (2006) | Biomechanics | Undergraduate | Quasi-exp | Hard question performance (CBI vs. Traditional) | 23 | NR | p = 0.022 | CBI group performed significantly better | Cognitive / Academic Achievement |
| 2 | 1 | Roselli & Brophy (2006) | Biomechanics | Undergraduate | Quasi-exp | Easy question performance (CBI vs. Traditional) | 27 | NR | p = 0.330 | No significant difference | Cognitive / Academic Achievement |
| 3 | 2 | Martín et al. (2007) | Biomedical Eng. | Undergraduate | Quasi-exp | Knowledge gain (HPL vs. Traditional) | NR | NR | p > 0.05 | No significant difference | Cognitive / Academic Achievement |
| 4 | 2 | Martín et al. (2007) | Biomedical Eng. | Undergraduate | Quasi-exp | Innovative thinking skills (HPL vs. Traditional) | NR | NR | p < 0.05 | HPL group significantly superior | Higher-Order Thinking |
| 5 | 3 | Jansen et al. (2003) | Biomedical Optics | Undergraduate | Pre-Post | Student engagement (Pre vs. Post) | NR | NR | p < 0.05 | Post-test score significantly improved | Learning Engagement |
| 6 | 3 | Jansen et al. (2003) | Biomedical Optics | Undergraduate | Pre-Post | Conceptual understanding (Pre vs. Post) | NR | NR | p < 0.05 | Post-test score significantly improved | Cognitive / Academic Achievement |
| 7 | 4 | Han (2011) | Biotransport | Undergraduate | Pre-Post | Academic achievement (CBL Pre vs. Post) | NR | NR | NR | Academic performance improved | Cognitive / Academic Achievement |
| 8 | 5 | Barr et al. (2004) | Biomechanics | Undergraduate | Quasi-exp | Academic performance (Exp vs. Con) | 16 / 16 (32) | NR | NR | Positive effect trend | Cognitive / Academic Achievement |
| 22 | 9 | Tang & Chow (2020) | Nursing | Undergraduate | Quasi-exp | Deep learning approach (Intervention Pre vs. Post) | 130 | NR | F = 5.2, p = 0.023 | Significant shift toward deep learning | Learning Strategies |
| 23 | 9 | Tang & Chow (2020) | Nursing | Undergraduate | Quasi-exp | Surface learning approach (Intervention Pre vs. Post) | 130 | NR | F = 2.4, p = 0.125 | Surface learning tendency decreased (non-sig.) | Learning Strategies |
| 27 | 12 | Félix-Herrán et al. (2022) | Engineering | Undergraduate | Case/Quasi | Professional competencies (Exp vs. Con) | NR | NR | p < 0.05 | UAV project competencies grew significantly | Comprehensive Competencies |
| 28 | 13 | Indonesian Researchers (2018) | Chemistry | Undergraduate | Quasi-exp | Creative thinking skills (CBL vs. PBL) | NR | NR | p < 0.05 | CBL score superior in creative thinking | Higher-Order Thinking |
| 35 | 15 | Hu et al. (2018) | Gastrointestinal Surg. | Undergraduate (Clerkship) | Quasi-exp | Theoretical knowledge (Obs vs. Con) | 25 / 25 (50) | NR | p > 0.05 | Group difference not statistically significant | Cognitive / Academic Achievement |
| 36 | 15 | Hu et al. (2018) | Gastrointestinal Surg. | Undergraduate (Clerkship) | Quasi-exp | Clinical practical operations (Obs vs. Con) | 25 / 25 (50) | NR | p < 0.05 | Micro-lecture + CBL group superior | Practical Skills |
| 37 | 15 | Hu et al. (2018) | Gastrointestinal Surg. | Undergraduate (Clerkship) | Quasi-exp | Teaching satisfaction (Obs vs. Con) | 25 / 25 (50) | NR | p < 0.05 | Teaching satisfaction significantly improved | Attitude & Satisfaction |
| 38 | 16 | Stomatology Researchers (2023) | Stomatology | Undergraduate | Quasi-exp | Satisfaction (Exp vs. Con) | 158 / 105 (263) | NR | p < 0.05 | Higher satisfaction in Exp group (2017 Cohort) | Attitude & Satisfaction |
| 39 | 16 | Stomatology Researchers (2023) | Stomatology | Undergraduate | Quasi-exp | Learning interest (Exp vs. Con) | 158 / 105 (263) | NR | p < 0.05 | Professional interest significantly increased | Attitude & Satisfaction |
| 40 | 17 | Li et al. (2016) | Nursing | Undergraduate | Quasi-exp | Exam performance (Exp vs. Con) | 34 / 34 (68) | NR | p < 0.05 | Interpersonal communication theory improved | Cognitive / Academic Achievement |
| 41 | 17 | Li et al. (2016) | Nursing | Undergraduate | Quasi-exp | Collaboration skills (Exp vs. Con) | 34 / 34 (68) | NR | p < 0.05 | Teamwork score increased | Collaboration & Communication |
| 42 | 17 | Li et al. (2016) | Nursing | Undergraduate | Quasi-exp | Autonomous learning ability (Exp vs. Con) | 34 / 34 (68) | NR | p < 0.05 | Self-directed learning metrics increased | Motivation & Self-Efficacy |
| 43 | 18 | Membrillo-Hernández et al. (2017) | Sustainable Eng. | Undergraduate | Quasi-exp | Deep learning & problem solving | 45 / 42 (87) | Me = 85.4, SD = 8.2; Mc =78.1, SD = 9.5 | t = 3.84, p < 0.001 | d = 0.82, CBL significantly enhanced deep learning competencies | Higher-Order Thinking / Deep Learning |
| 44 | 20 | Suwono et al. (2019) | Biology | Undergraduate | Quasi-exp | Scientific literacy (Exp vs. Con) | 31 / 29 (60) | NR | p < 0.05 | CBL higher than PBL and lecture groups | Overall / Scientific Literacy |
| 45 | 21 | Data Mining Study (2019) | Data Mining | Undergraduate | Pre-Post | Comprehensive lab performance (Pre vs. Post) | NR | NR | p < 0.05 | Data mining coding capability enhanced | Practical Skills |
| 46 | 22 | Medical Inform. (2018) | Medical Informatics | Undergraduate | Pre-Post | Interest & hands-on capability (Pre vs. Post) | NR | NR | p < 0.05 | Practical project completion rate was high | Practical Skills |
| 47 | 1 | Roselli & Brophy (2006) | Biomechanics | Undergraduate | Descriptive | Hard question perf.—35% CBI superior | 23 | — | — | 35% of items performed significantly better | Cognitive / Percentage |
| 48 | 1 | Roselli & Brophy (2006) | Biomechanics | Undergraduate | Descriptive | Hard question perf.—4% Trad. superior | 23 | — | — | 4% of items traditional instruction better | Cognitive / Percentage |
| 49 | 1 | Roselli & Brophy (2006) | Biomechanics | Undergraduate | Descriptive | Hard question perf.—61% No diff. | 23 | — | — | 61% of items performance equal across groups | Cognitive / Percentage |
| 50 | 5 | Barr et al. (2004) | Biomechanics | Undergraduate | Quasi-exp | Pre-test performance (Exp vs. Con) | 16 / 16 (32) | NR | NR | No baseline statistical difference between groups | Cognitive / Academic Achievement |
| 51 | 5 | Barr et al. (2004) | Biomechanics | Undergraduate | Quasi-exp | Post-test performance (Exp vs. Con) | 16 / 16 (32) | NR | NR | Exp group post-test mean higher than Con | Cognitive / Academic Achievement |
| 52 | 5 | Barr et al. (2004) | Biomechanics | Undergraduate | Quasi-exp | Perceived learning effect (Exp vs. Con) | 16 / 16 (32) | NR | NR | Positive student satisfaction with modules | Attitude & Satisfaction |
| 63 | 9 | Tang & Chow (2020) | Nursing | Undergraduate | Quasi-exp | Deep learning approach—F statistic | 130 | — | F = 5.2, p = 0.023 | ANOVA significant | Test Statistic |
| 64 | 9 | Tang & Chow (2020) | Nursing | Undergraduate | Quasi-exp | Surface learning approach—F statistic | 130 | — | F = 2.4, p = 0.125 | ANOVA non-significant | Test Statistic |
| 70 | 15 | Hu et al. (2018) | Gastrointestinal Surg. | Undergraduate | Quasi-exp | Theoretical knowledge—Obs vs. Con | 25 / 25 (50) | NR | p > 0.05 | No statistical difference in theory scores | Cognitive / Academic Achievement |
| 71 | 15 | Hu et al. (2018) | Gastrointestinal Surg. | Undergraduate | Quasi-exp | Clinical operations—Obs vs. Con | 25 / 25 (50) | NR | p < 0.05 | Practical skill advantage significant | Practical Skills |
| 72 | 15 | Hu et al. (2018) | Gastrointestinal Surg. | Undergraduate | Quasi-exp | Excellent/good pass rate—Obs vs. Con | 25 / 25 (50) | NR | NR | Observation group had higher excellent rate | Cognitive / Academic Achievement |
| 73 | 16 | Stomatology Researchers (2023) | Stomatology | Undergraduate | Quasi-exp | Satisfaction—Exp vs. Con | 158 / 105 (263) | NR | p < 0.05 | Satisfaction difference significant | Attitude & Satisfaction |
| 74 | 16 | Stomatology Researchers (2023) | Stomatology | Undergraduate | Quasi-exp | Learning interest—Exp vs. Con | 158 / 105 (263) | NR | p < 0.05 | Interest improvement difference significant | Attitude & Satisfaction |
| 75 | 17 | Li et al. (2016) | Nursing | Undergraduate | Quasi-exp | Exam performance—Exp vs. Con | 34 / 34 (68) | NR | p < 0.05 | Academic performance increased | Cognitive / Academic Achievement |
| 76 | 17 | Li et al. (2016) | Nursing | Undergraduate | Quasi-exp | Collaboration skills—Exp vs. Con | 34 / 34 (68) | NR | p < 0.05 | Teamwork score significantly higher | Collaboration & Communication |
| 77 | 17 | Li et al. (2016) | Nursing | Undergraduate | Quasi-exp | Autonomous learning ability—Exp vs. Con | 34 / 34 (68) | NR | p < 0.05 | Autonomous learning significantly higher | Motivation & Self-Efficacy |
| 78 | 18 | Fan & Li (2023) | Higher Education | College Students | Meta-Analysis | Deep learning—Meta-analytic overall effect | — | — | p < 0.05 | Overall effect size significantly positive | Overall / Meta-Analysis |

Notes:

*N*_exp_ / *N*_con_ (*N*_total_): Sample size for Experimental / Control groups (Total sample size).

NR: Not Reported; -: Not Applicable / Dimensionless metric.

Outcome Domain classifications map directly to the systematic framework (Cognitive, High-Order Thinking, Motivation & Self-Efficacy, Engagement, Affect, Practical Skills, Collaboration & Communication, Attitude & Satisfaction, Overall).

**References for Primary Included Studies**

(Note: Studies included in the primary meta-analysis synthesis are marked with an asterisk *)

*Barr, R., Pandy, M., & Petrosino, A. (2004). Classroom testing of VaNTH biomechanics learning modules. In Proceedings of the 2004 Annual Conference (pp. 9.311.1–9.311.20).

Membrillo-Hernández, J., Ramírez-Cadena, M. de J., Caballero-Valdés, C., et al. (2017). Challenge based learning: The case of sustainable development engineering at the Tecnologico de Monterrey, Mexico City Campus. In Proceedings of the International Conference on Interactive Collaborative Learning (pp. 908–914). Springer International Publishing.

*Félix-Herrán, L. C., Izaguirre-Espinosa, C., Parra-Vega, V., et al. (2022). A challenge-based learning intensive course for competency development in undergraduate engineering students: Case study on UAVs. Electronics, 11(9), Article 1349.

*Han, B. (2011). Modified challenge-based teaching of biotransport phenomena. In Summer Bioengineering Conference (Vol. 54587, pp. 753–754). American Society of Mechanical Engineers.

*Jansen, E. (2003). Implementation and assessment of challenge based instruction in a biomedical optics course. In Proceedings of the 2003 Annual Conference (pp. 8.656.1–8.656.8).

Leijon, M., Gudmundsson, P., Staaf, P., et al. (2022). Challenge based learning in higher education–A systematic literature review. Innovations in Education and Teaching International, 59(5), 609–618.

*Li, Y. R., Jin, R. H., Zheng, J., et al. (2016). Exploration of the application effect of challenge-based learning in nursing interpersonal communication course. Chinese Medical Innovation, 13(13), 75–78.

*Martin, T., Rivale, S. D., & Diller, K. R. (2007). Comparison of student learning in challenge-based and traditional instruction in biomedical engineering. Annals of Biomedical Engineering, 35(8), 1312–1323.

*Hu, G., & Sun, J. C. (2018). Application effect of micro-lecture combined with challenge-based learning in gastrointestinal surgery clerkship teaching. China Continuing Medical Education, 10(16), 5–7.

*Roselli, R. J., & Brophy, S. P. (2006). Effectiveness of challenge-based instruction in biomechanics. Journal of Engineering Education, 95(4), 311–324.

*Stomatology Education Research Group. (2023). Design and application of challenge-based learning teaching model in stomatology education: Taking Pediatric Dentistry as an example. Journal of Modern Medicine & Health, 39(11), 1943–1946.

*Suwono, H., Saefi, M., & Susilo, H. (2019). Challenge based learning to improve scientific literacy of undergraduate biology students. AIP Conference Proceedings, 2081(1), Article 030020.

*Tang, A. C. Y., & Chow, M. C. M. (2020). To evaluate the effect of challenge-based learning on the approaches to learning of Chinese nursing students: A quasi-experimental study. Nurse Education Today, 85, Article 104293.

**Reproducible R Script for Manuscript & OSF Repository**

```
==============================================================================
# Script: Three-Level Meta-Analysis and Robust Variance Estimation (RVE)
# Package Dependencies: metafor, clubSandwich, dplyr
#
==============================================================================

# 1. Load required libraries
library(metafor)
library(clubSandwich)
library(dplyr)

# 2. Load dataset (Example Structure)
# Expected columns: study_id, effect_id, g (Hedges' g), vg (sampling variance)
dat <- read.csv("Meta_Analysis_Dataset.csv")

# 3. Step 1: Construct Approximate Variance-Covariance Matrix (V)
# Assumes within-study correlation r = 0.60 among dependent effect sizes
V_mat <- vcalc(vi = vg, cluster = study_id, rho = 0.60, data = dat)

# 4. Step 2: Fit Three-Level Random-Effects Model using REML
res_3level <- rma.mv(
  yi = g,
  V = V_mat,
  random = ~ 1 | study_id / effect_id,
  data = dat,
  method = "REML",
  sparse = TRUE
)

# Display standard three-level model output
summary(res_3level)

# Calculate Variance Distribution across Levels (Cheung, 2014)
total_var <- res_3level$sigma2[1] + res_3level$sigma2[2] + mean(dat$vg)
pct_l1 <- (mean(dat$vg) / total_var) * 100
pct_l2 <- (res_3level$sigma2[2] / total_var) * 100 # Within-study variance
pct_l3 <- (res_3level$sigma2[1] / total_var) * 100 # Between-study variance

cat(sprintf("Variance Distribution:\n Level 1 (Sampling Error): %.2f%%\n Level 2 (Within-Study): %.2f%%\n Level 3 (Between-Study): %.2f%%\n",
  pct_l1, pct_l2, pct_l3))

# 5. Step 3: Apply Cluster-Robust Variance Estimation (RVE) with CR2 Adjustment
# Uses Satterthwaite degrees of freedom correction
rve_results <- coef_test(
  res_3level,
  vcov = "CR2",
  cluster = dat$study_id,
  test = "Satterthwaite"
)

rve_ci <- conf_int(
  res_3level,
  vcov = "CR2",
  cluster = dat$study_id,
  test = "Satterthwaite"
)

# Print Final Robust Results
cat("\n--- Cluster-Robust Variance Estimation (CR2 / Satterthwaite) Results ---\n")
print(rve_results)
print(rve_ci)

# 6. Step 4: Robust Moderator / Subgroup Analysis Example
# Example for categorical moderator 'discipline'
mod_model <- rma.mv(
  yi = g,
  V = V_mat,
  mods = ~ factor(discipline) - 1,
  random = ~ 1 | study_id / effect_id,
  data = dat,
  method = "REML"
)

# RVE Test for Moderator Differences (Omnibus Test)
mod_rve <- Wald_test(
  mod_model,
  constraints = constrain_zero(1:length(unique(dat$discipline))),
  vcov = "CR2",
  cluster = dat$study_id,
  test = "Satterthwaite"
)

print(mod_rve)
```
